# Benzofuran Derivatives from Cortex Mori Radicis and Their Cholinesterase-Inhibitory Activity

**DOI:** 10.3390/molecules29020315

**Published:** 2024-01-08

**Authors:** Xiang Cui, Zehong Huang, Shanshan Deng, Yunxia Zhang, Guoyin Li, Lining Wang, Yanru Deng, Changjing Wu

**Affiliations:** 1 College of Life Sciences and Agronomy, Zhoukou Normal University, Zhoukou 466001, China; cuixiang921@126.com (X.C.);; 2 College of Traditional Chinese Medicine, Tianjin University of Traditional Chinese Medicine, Tianjin 301617, China; 3 Field Observation and Research Station of Green Agriculture in Dancheng County, Zhoukou 466001, China

**Keywords:** Cortex Mori, benzofuran derivatives, structure elucidation, butyrylcholinesterase inhibitor

## Abstract

The phytochemical investigation of Cortex Mori Radicis led to the isolation and identification of a new prenylated benzofuranone (**1**) and four ring-opening derivatives (**2**–**5**) named albaphenol A–E, as well as nigranol A (**6**), together with ten 2-arylbenzofuran derivatives (**7**–**16**). The characterization of the structures of the new compounds and the structural revision of nigranol A (**6**) were conducted using the comprehensive analysis of spectroscopic data (1D/2D NMR, HRESIMS, CD, and XRD). Compounds **1**–**16** were tested for their inhibitory effects on acetylcholinesterase (AChE) and butyrylcholinesterase (BChE). Compounds **1** and **4** showed weak BChE-inhibitory activity (IC_50_ 45.5 and 61.0 μM); six 2-arylbenzofuran derivatives showed more-potent BChE-inhibitory activity (IC_50_ 2.5–32.8 μM) than the positive control galantamine (IC_50_ 35.3 μM), while being inactive or weakly inhibitory toward AChE. Cathafuran C (**14**) exhibited the most potent and selective inhibitory activity against BChE in a competitive manner, with a Ki value of 1.7 μM. The structure–activity relationships of the benzofuran-type stilbenes were discussed. Furthermore, molecular docking and dynamic simulations were performed to clarify the interactions of the inhibitor–enzyme complex.

## 1. Introduction

Alzheimer’s disease (AD) is the most common and ultimately fatal degenerative brain disorder, characterized by central cognitive and behavioral deficits (2021 Alzheimer’s disease facts and figures). According to the World Alzheimer Report 2019, there are over 50 million AD patients worldwide today, and this number is expected to increase to 152 million by 2050 [1]. Several hypotheses have been developed to explain the pathogenesis and progression of AD, such as amyloid-β (Aβ) deposits [2], tau protein aggregation [3], neuroinflammation [4], mitochondrial dysfunction [5], cholinergic dysfunction [6], etc. The cholinergic hypothesis suggests that in AD pathological conditions, cholinergic neurons are extensively damaged and die, and the activity of choline acetyltransferase is significantly reduced. As a result, the level of acetylcholine (ACh) in the brains of AD patients continues to decrease, leading to impairments in learning and memory function [6]. Therefore, increasing the levels of ACh in the brains of people with Alzheimer’s disease can effectively improve their cognitive statuses. One way to increase ACh levels is to inhibit cholinesterase, which is responsible for catalyzing the hydrolysis of ACh. There are two kinds of ChEs in the body, namely, acetylcholinesterase (AChE, EC 3.1.1.7) and butyrylcholinesterase (BChE, EC 3.1.1.8). Donepezil, galantamine, and rivastigmine are all AChE inhibitors used in the clinical treatment of AD. However, in the brains of moderate to severe AD patients, BChE replaces AChE as the main metabolic enzyme of ACh. Therefore, inhibiting BChE at this stage can more effectively increase ACh levels, and the BChE inhibitor has been regarded as a potential therapeutic agent for AD [7]. In the last decade, the discovery of potent and selective BChE inhibitors has inspired many efforts, and many molecules have been identified as excellent BChE inhibitors with good activity toward AD treatment in animal models [8,9,10]. Natural products (NPs) and their derivatives also play remarkable roles in finding novel BChE inhibitors [11,12,13,14]. In our recent study on BChE-inhibitory NPs, we discovered that butenolide derivatives from *Aspergillus terreus* selectively inhibit BChE in competitive manners [15].

Cortex Mori Radicis (Sangbaipi), an important Chinese herbal medicine officially listed in the Chinese Pharmacopoeia, is the root bark of some Morus species (particularly *M. alba*) [16] and has a long history of use as antidiabetic, diuretic, and expectorant agents in traditional Chinese medicine. Diverse groups of phytochemicals have been isolated from Cortex Mori Radicis, such as Diels–Alder-type adducts, stilbenes, flavonoids, and alkaloids [17,18]. Many of these compounds exhibit various biological activities, including antioxidant, anti-inflammatory, antimicrobial, antitumor, anti-hypotensive, and antidiabetic [17,18,19]. As for anti-Alzheimer’s disease activity, several kinds of chemical constituents have exhibited the potential function of targeting multiple AD-related enzymes. For example, moracin derivatives were discovered to function as dual inhibitors of BACE1 and cholinesterase, and moracin S showed potent inhibition of BChE [20]. In addition, several arylbenzofurans from the root bark of Morus alba functioned as triple inhibitors of cholinesterase, BACE1, and GSK-3β, and mulberrofuran D and D2 strongly inhibited BChE with IC_50_ values of 6.12 and 1.51 μM [21]. Moreover, Diels−Alder-type adducts have been characterized as multitargeted agents for Alzheimer’s disease; mulberrofuran G and albanol B showed strong AChE- and BChE-inhibitory activities [22,23]. These results suggest that the root bark of Morus alba is an important source of potent BChE-inhibitory NPs. In the present study, we isolated and identified sixteen benzofuran derivatives from Cortex Mori Radicis, including five new compounds and ten 2-arylbenzofuran derivatives. The ChEs’ inhibitory activities were evaluated to screen for potent BChE inhibitors. Herein, we report the isolation, structural determination, and enzyme inhibition evaluation of the isolated compounds and the molecular docking and dynamic simulation of inhibitor–enzyme interactions.

## 2. Results and Discussion

### 2.1. Structure Determination of Compounds ***1**–**16***

**Figure 1:**
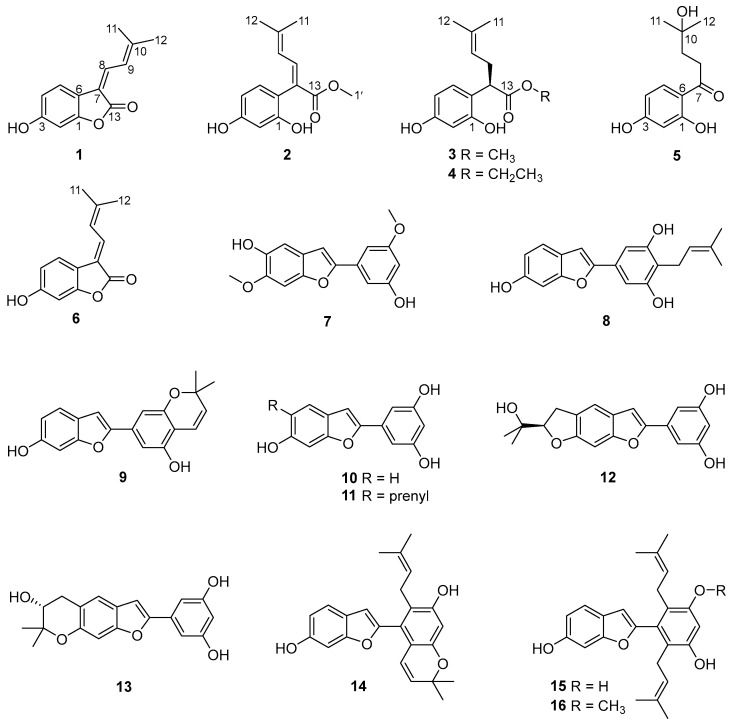
Structures of compounds **1**–**16** isolated from Cortex Mori Radicis.

Sixteen compounds were isolated from a partition eluted with 80% ethanol using AB-8 macroporous resin column chromatography of the ethanolic extract Cortex Mori Radicis. Based on a comprehensive analysis of various spectroscopic data including high-resolution electrospray ionization mass spectra (HRESIMS), 1D/2D nuclear magnetic resonance (NMR) spectra, and ultraviolet (UV) spectra, together with X-ray single-crystal diffraction, five previously undescribed compounds were elucidated and named albaphenols A–E (**1**–**5**), and eleven known compounds were identified, namely, nigranol A (**6**) [24], moracin B (**7**) [25], moracin C (**8**) [26], moracin D (**9**) [26], moracin M (**10**) [27], moracin N (**11**) [28], moracin O (**12**) [29], moracin P (**13**) [29], cathafuran C (**14**) [30], mulberrofuran V (**15**) [31], and mulberrofuran N (**16**) [32], via comparison with NMR data in the literature (Figure 1). 

**Figure 2:**
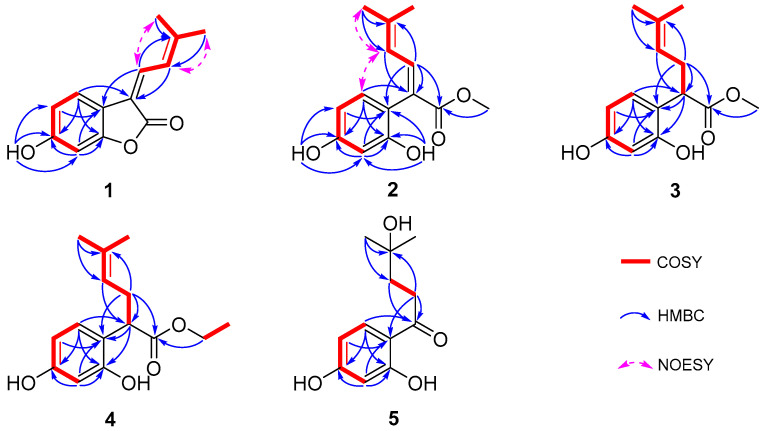
Key ^1^H-^1^H COSY, HMBC, and NOESY correlations of compounds **1**–**5**.

Compound **1** was obtained as a yellow solid, and its molecular formula was determined to be C_13_H_12_O_3_ via HRESIMS (*m*/*z* 215.0703 [M-H]^−^, calculated for 215.0708), indicating eight degrees of unsaturation. According to the distortionless enhancement using polarization transfer (DEPT) and heteronuclear single-quantum correlation (HSQC) spectra, the ^13^C NMR spectrum showed 13 carbon signals, including two methyl groups, five sp^2^-methines, and six quaternary carbons. The ^1^H NMR spectroscopic data of **1** (Table 1) showed a series of proton signals and were determined to be affiliated with the relevant carbons using HSQC analysis. An aromatic ABX spin system operated at δ_H_ 7.54 (1H, d, *J* = 8.3 Hz, H-5), 6.65 (1H, dd, *J* = 8.3, 2.2 Hz, H-4), and 6.65 (1H, d, *J* = 2.2 Hz, H-2) in the ^1^H NMR spectrum suggested a 1,3,4- or 1,3,6-trisubstituted benzene ring. Additionally, the signals of two adjacent coupling olefinic protons at δ_H_ 7.64 (1H, d, *J* = 12.8 Hz, H-8) and 7.39 (1H, br d, *J* = 12.2, H-9) and two methyl groups linked to olefinic carbon at δ_H_ 2.04 and 2.01 (each 3H, br s, H-11,12) were observed in the ^1^H NMR spectrum, and the H-H-correlated spectroscopy (H-H COSY) correlation between olefinic proton at δ_H_ 7.39 and the two methyl groups suggested a fragment of -C=CH-CH=C(CH_3_)_2_, which was further confirmed by the heteronuclear multiple bond correlation (HMBC) signals from H-9 to C-7, C-11, and C-12 and from H-8 and H-11/12 to C-10. Moreover, the HMBC signals from H-8 to C-6 and from H-5 to C-7 indicated the above fragment was linked to the benzene ring at C-6, and a hydroxyl group at C-3 was identified through the HMBC correlation between a phenolic hydroxyl group δ_H_ 8.93 (br s, 1H) and C-3 (Figure 2). Meanwhile, the HMBC signal from H-8 to the carbonyl C-13 (δ_C_ 167.8) indicated the linkage of C-7 with C-13. Another oxygenated olefinic carbon signal at δ_C_ 154.9 (C-1) and one remaining degree of unsaturation led to the suggestion that C-13 is linked to C-1 via an oxygen atom forming a benzofuranone core. Consequently, compound **1** was identified as 6-hydroxy-3-(3-methylbut-2-en-1-ylidene)benzofuran-2(3*H*)-one, with the same planar structure as compound **6**, suggesting a pair of geometric isomers for **1** and **6**, and the structural difference was the configuration of a Δ_7,8_ double bond. 

**Figure 3:**
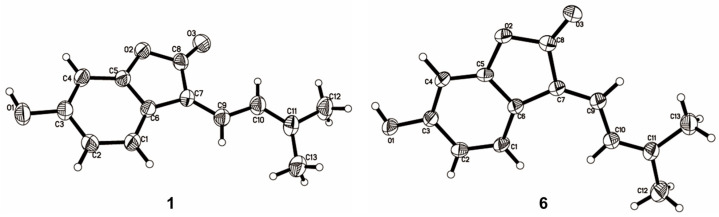
ORTEP (Oak Ridge thermal ellipsoid plot program) plots of the X-ray crystal structures of compounds **1** and **6**.

The NMR data of **6** (Table 1 and Table 2) were identical to those of nigranol A previously isolated from *Morus nigra* Linn [24]. The Δ_7,8_ double bond of nigranol A was formerly determined to be a *Z*-configuration according to the relatively large coupling constant (12.8 Hz) between H-8 and H-9, which is not applicable and convincing in view of the freely rotating single bond between C-8 and C-9. Hence, the configurations of the Δ_7,8_ double bonds for **1** and **6** are still ambiguous. However, it was not possible to determine the configuration of the Δ_7,8_ double bond using NOESY correlations for H-8 or H-9 with H-5 since no relative signals were observed in the ROESY spectrum of **1** (Supplementary Materials). Fortunately, the crystals of compounds **1** and **6** were obtained, and single-crystal X-ray diffraction (XRD) analyses were conducted to determine the configuration of the Δ_7,8_ double bond. As a result, the isobutene moiety (C-9) and the carbonyl group (C-13), the larger substituent groups for C-8 and C-7, respectively, existed in *cis*- and *trans*-conformations in compounds **1** and **6**, respectively (Figure 3). Therefore, compound **1** was determined to be (*Z*)-6-hydroxy-3-(3-methylbut-2-en-1-ylidene)benzofuran-2(3H)-one and named albaphenol A, while nigranol A (**6**) was revised as (*E*)-6-hydroxy-3-(3-methylbut-2-en-1-ylidene)benzofuran-2(3H)-one. Interestingly, compounds **1** and **6** could change into each other, and this *cis*-*trans*-tautomerism could be promoted using UV exposure at 254 and 365 nm, which almost achieved equilibrium (approximately in a ratio of 1:1) within 30 min of photoisomerization (Figure S1 in Supplementary Materials). Due to the *cis*–*trans* tautomerism also taking place slowly under natural light, the samples were stored in a 4 °C refrigerator under dark conditions, and the crystallizations of the two compounds were induced in nearly saturated methanol solution under the same conditions.

Compound **2** was purified as a yellow solid; its molecular formula was established as C_14_H_16_O_4_ on the basis of an HRESIMS peak at *m*/*z* 249.1124 [M+H]^+^ (calcd. for C_14_H_17_O_4_ 249.1122), with an index of hydrogen deficiency of seven. The presence of a 1,3,6-trisubstituted benzene ring was indicated by three aromatic hydrogen signals at δ_H_ 6.80 (1H, d, *J* = 8.2 Hz, H-5), 6.42 (1H, d, *J* = 2.4 Hz, H-2), and 6.37 (1H, dd, *J* = 8.2, 2.4 Hz, H-4), whose coupling relationships were confirmed using COSY analysis (Figure 2). Furthermore, two adjacent coupling olefinic protons at δ_H_ 7.60 (1H, d, *J* = 11.8 Hz, H-8) and 5.86 (1H, br d, *J* = 11.8 Hz, H-9) together with two methyl signals at δ_H_ 1.91 and 1.79 suggested that compound **2** had the same isopentenyl moiety as **1**, which was verified using extensive HSQC and HMBC analyses. Additionally, there were two phenolic hydroxyl signals at δ_H_ 7.97 and 8.26, which were linked to C-1 and C-3 of the benzene ring, respectively, according to the HMBC correlations (Figure 2). Moreover, a methoxy group was deduced from the signals at δ_H_ 3.64 (3H, s, H-1′) and δ_C_ 51.8 (C-1′), which was further appointed to a carbonyl according to the HMBC correlation of H-1′ and C-13 (δ_C_ 177.3). The HMBC signals from H-8 to C-6 and C-13 and H-5 to C-7 indicated the linkage of C-7 with C-6 and C-13, respectively. Taken together, compound **2** was assumed to be the methanolysis product of **6** with an *E*-configuration of the Δ_7,8_ double bond according to the NOESY correlations for H-9 and H-5 (Figure 1 and Figure 2) and named albaphenol B.

Compound **3**, obtained as a white and amorphous solid, was identified as C_14_H_18_O_4_ from the quasi-molecular ion peak [M-H]^−^ at *m*/*z* 249.1126 (calcd. for C_14_H_17_O_4_ 249.1127) in its HRESIMS spectrum together with its ^13^C NMR spectrum, indicating two more hydrogen and one degree of unsaturation less than compound **2**. Upon analyzing the ^1^H NMR and COSY spectra of **3**, some structural differences from **2** were found, such as an sp^3^ hybrid methene at δ_H_ 2.59 (1H, ddd, *J* = 14.6, 8.0, 7.3 Hz, Ha-8) and 2.37 (1H, dt, *J* = 14.6, 7.3 Hz, Hb-8) and an sp^3^ hybrid methine at δ_H_ 3.85 (1H, dd, *J* = 8.0, 7.3, H-7), instead of the Δ_7,8_ double bond signals; additionally, a coupling relationship was found between H-9 (δ_H_ 5.08, br t, *J* = 7.3 Hz) and the methine (Table 1 and Figure 2). Consistently, the ^13^C NMR and DEPT spectra exhibited sp^3^ carbon signals at δ_c_ 45.6 (C-7) and δ_c_ 32.1 (C-8) instead of the two olefinic carbons signals for compound **2** (Table 2). The above information allowed us to suppose that compound **3** was the hydrogenation derivative of compound **2** at the Δ_7,8_ double bond. Extensive analyses of the 1D and 2D NMR spectra confirmed the structure of compound **3** to be methyl 2-(2,4-dihydroxyphenyl)-5-methylhex-4-enoate (Figure 1), which was named albaphenol C. There is one chiral center at C-7 in compound **3**, and its absolute configuration was determined according to its optical properties. The structural analogs of compound **3** possessing an *R*-configuration, replacing the benzene ring with different substituted phenyl groups or even other types of aromatic nuclei, all exhibited levorotation at 589.3 nm [33]. Therefore, the absolute stereochemistry of compound **3** at C-7 was determined to be an *R*-configuration according to its negative specific optical rotation ([α]_D_) value of −42.0, which was further supported by the negative Cotton effect at 200–220 nm in the circular dichroism (CD) spectrum of compound **3** (Supplementary Materials) [34].

Compound **4** was also obtained as a white amorphous powder, and its HRESIMS spectrum provided the quasi-molecular ion peak [M-H]^−^ at *m*/*z* 263.1282 (calcd. for C_15_H_19_O_4_ 263.1283), establishing the molecular formula as C_15_H_20_O_4_, which is one more CH_2_ than that of compound **3**. Compound **4** has very similar NMR data to **3**, except that an ethoxy group was observed, corresponding to δ_H_ 4.09 and 4.07 (each 1H, dq, *J* = 16.6, 7.1 Hz, H-1′) and δ_H_ 1.18 (3H, t, *J* = 7.1 Hz, H-2′), according to the COSY correlation (Figure 2). The ethoxy group was further linked to the carbonyl based on the HMBC correlation from H-1′ to C-13 (δ_C_ 176.9). Finally, compound **4** was identified as ethyl 2-(2,4-dihydroxyphenyl)-5-methylhex-4-enoate using comprehensive analyses of 1D and 2D NMR spectra and named albaphenol D (Figure 1). The chiral center C-7 of compound **4** was also assumed to be in an *R*-configuration based on its negative [α]_D_ value of −15.3 and the negative Cotton effect at 200–220 nm (Supplementary Materials). 

The molecular formula of compound **5** was established as C_12_H_16_O_4_ according to the quasi-molecular ion peak at *m*/*z* 223.0966 ([M-H]^−^, calcd. for C_12_H_15_O_4_ 223.0970) in the HRESIMS spectrum, indicating five degrees of unsaturation. The ^1^H NMR spectra exhibited three aromatic hydrogen signals at δ_H_ 6.25 (1H, d, *J* = 2.4 Hz, H-2), 6.37 (1H, dd, *J* = 8.8, 2.4 Hz, H-4), and 7.77 (1H, d, *J* = 8.8 Hz, H-5); two coupling methylene signals at δ_H_ 3.10–2.93 (2H, m, H-8) and 1.96–1.76 (2H, m, H-9); and a gem-dimethyl signal at δ_H_ 1.24 (6H, s, H-11, 12). Based on the analyses of the DEPT and HSQC spectra, the ^13^C NMR spectrum showed twelve carbon signals, including two methyls with chemical equivalence at δ_C_ 29.2 (C-11 and C-12); two methylenes at δ_C_ 34.0 and 39.1 (C-8 and C-9); three sp^2^ methines at δ_C_ 103.7 (C-2), δ_C_ 109.1 (C-4), and δ_C_ 133.7 (C-5); and two oxygenated olefinic carbon signals at δ_C_ 166.4 (C-1) and δ_C_ 166.5 (C-3), along with an olefinic carbon signal at δ_C_ 113.9 (C-6), a carbonyl carbon signal at δ_C_ 206.5 (C-7), and an oxygenated sp^3^ tertiary carbon at δ_C_ 70.9 (C-10). The extensive HMBC analysis conducted suggested that compound **5** possessed the same 1,3,6-trisubstituted benzene moiety as compounds **1**–**4** (Figure 2). The HMBC signals from H-11 to C-10 and C-9 and H-8 to C-10 indicated a hydrated isopentenyl, which was further linked to the C-6 of the benzene ring via the carbonyl C-7 according to the HMBC correlations between H-5, H-8, and H-9 and C-7 (Figure 2). Consequently, compound **5** was identified as 1-(2,4-dihydroxyphenyl)-4-hydroxy-4-methylpentan-1-one and named albaphenol E. From a structural point of view, compound **5** was assumed to be derived from compound **3** through hydrolyzation, decarboxylation, oxidation, and hydration reactions.

### 2.2. ChE-Inhibitory Activities

Compounds **1**–**16** isolated from Cortex Mori Radicis were subjected to AChE- and BChE-inhibitory-activity tests. As shown in Table 3, the new compound **1** showed a moderate inhibitory effect on BChE with an IC_50_ value of 45.5 μM, while the trans isomer nigranol A (**6**) weakly inhibited BChE (IC_50_ = 94.8 μM), suggesting that the cis configuration represented a preferred conformation interacting with BChE. However, the ring-opening derivatives **2**–**5** did not exhibit a significant effect on the BChE activity, except that **4** slightly inhibited BChE with an IC_50_ value of 61.0 μM. The ten benzofuran-type stilbenes (**7**–**16**) exhibited significant variation in BChE-inhibitory activities based on structural diversity. Moracin B (**7**) and M (**10**) without isopentenyl did not inhibit BChE at 100 μM, while the prenylated derivatives moracin C (**8**) and N (**11**) displayed potent inhibitory activity with IC_50_ values of 27.9 μM and 13.5 μM, respectively, even though the prenylation took place at a different benzene ring. Furthermore, compared to **8**, the isopentenyl in moracin D (**9**) was cyclized with the ortho-hydroxyl forming an α-chromene group, which led to enhanced BChE-inhibitory activity (IC_50_ = 9.5 μM). However, when the cyclized isopentenyl was further hydrated, the molecules seemed to be incapable of inhibiting BChE; for example, neither moracin O (**12**) nor P (**13**) exhibited a significant inhibitory effect on BChE even at 100 μM. Most notably, cathafuran C (**14**) possessed potent BChE-inhibiting activity, with an IC_50_ value of 2.6 μM, whereas the other two di-prenylated analogs without the cyclization of isopentenyl, mulberrofuran V (**15**) and mulberrofuran N (**16**) moderately inhibited BChE, with IC_50_ values of 27.1 and 32.8 μM, showing that methylation at the phenolic-OH has little effect on inhibitory potency, and the lowered efficiency of **15** and **16** may be due to the nonrigid dual isopentenyls with a bulky spatial structure. The inhibition activities toward BChE of all the above compounds showed different levels of selectivity. Among all the compounds, only **9** and **11** showed moderate inhibitory effects on AChE, with IC_50_ values of 81.2 μM and 40.5 μM, respectively, and the other ones did not inhibit AChE even at a concentration of 100 μM. Notably, compound **14** selectively inhibited BChE, with a selective index (AChE IC_50_/BChE IC_50_ ratio) of over 38. Among the tested compounds, moracin M (**10**), O (**12**), and P (**13**) were evaluated for their inhibitory activities against BChE in previous research [19] and exhibited moderate inhibitory effects with IC_50_ values of 38.08, 28.22, and 37.96 μM, respectively. The differences in the outcomes of the bioassays between the previous and present research may be attributed to the heterogeneity of enzyme activity or compound samples. However, the enhancement effect regarding BChE-inhibitory activity contributed by the isopentenyl group was also embodied by the more potent inhibition of BChE by moracin S [19].

**Figure 4:**
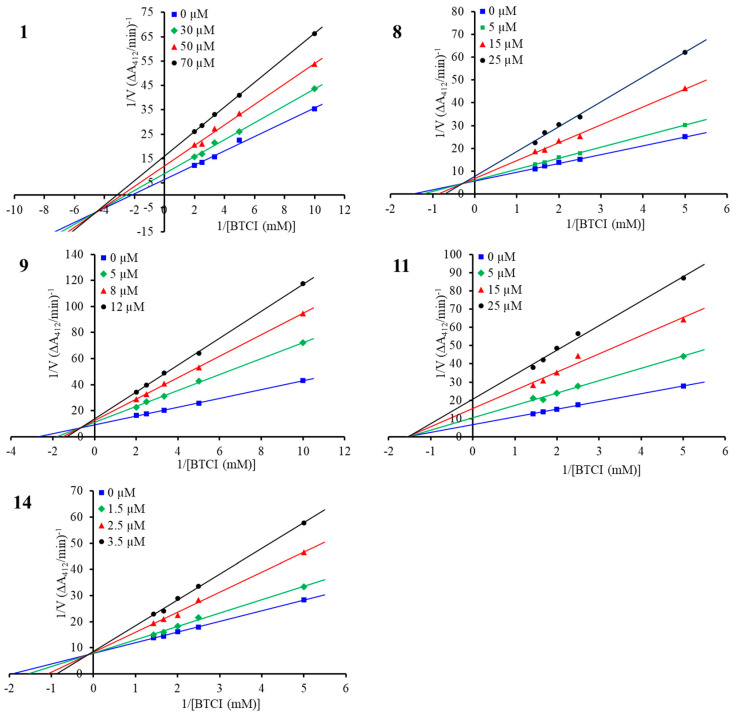
Lineweaver–Burk plot for BChE inhibition by compounds **1**, **8**, **9**, **11**, and **14**.

To determine the inhibition modes of the compounds, enzyme kinetic studies were carried out for the structurally representative inhibitors, namely, compounds **1**, **8**, **9**, **11**, and **14**. As shown in Figure 4, in the Lineweaver–Burk double-reciprocal plots, the plots of 1/V versus 1/[S] each provide a group of straight lines with different slopes that intersect at the third quadrant for compound **1** and at the second quadrant for the other four inhibitors, suggesting that they are all mixed-type inhibitors [35]. For this type of interaction, inhibitors can bind to the free enzyme (E) and the enzyme–substrate (ES) complex, forming EI and ESI complexes, respectively. The Ki and αKi values for the inhibitors were determined from the secondary plots of the slope (Km/Vm) and the vertical intercept (1/Vm) from the Lineweaver–Burk plot as functions of the inhibitor concentration, respectively (Figure S2 in Supplementary Materials), and the thermodynamic cooperativity factor α differed among the five structurally diverse inhibitors (Table 3) [35,36]. Compound **1** showed an α value of 0.46, suggesting **1** may engage in preferential binding to the ES complex in a mixed uncompetitive manner (α < 1). In contrast, compounds **8** and **9**, with α values of 5.55 and 4.42, showed greater affinity to E than to the ES complex, with this behavior being defined as a mixed competitive manner (α > 1). As for compound **11**, the inhibitor exhibited almost equivalent affinities to the E and ES complexes, with an α value of 1.05, approximately suggesting a noncompetitive mode of inhibitor interaction (α = 1). Notably, the most potent inhibitor, **14**, showed an α value of 13.35, indicating that the inhibitor mainly bound to E, which could be considered a competitive inhibitor of BChE (α > 10) [36].

### 2.3. Molecular Docking and Dynamics Simulation for Cathafuran C with BChE

**Figure 5:**
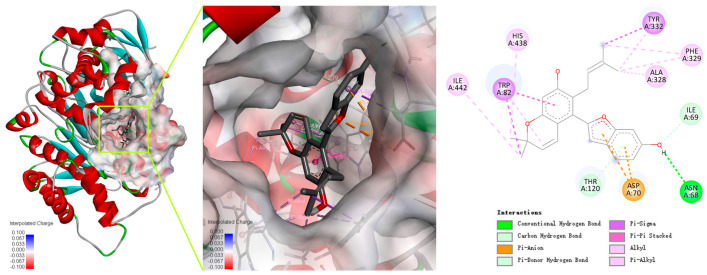
Predicted dock conformation and 2D interaction diagram between 5k5e and cathafuran C (**14)**.

Cathafuran C (**14**) potently inhibits BChE in a competitive manner and with quite high selectivity. The interaction modes of the inhibitor with the enzymes were investigated using Autodock Vina software in Yinfo Cloud Platform (http://cloud.yinfotek.com/) to better understand the capacity and mechanism regarding **14** binding with BChE. As a result, **14** could successfully insert itself into the binding groove of BChE, forming several kinds of interactions with the residues of the enzyme (Figure 5). The amino acid residue Trp82 engages in a π–π stacked interaction, a π–σ interaction, and a π–alkyl interaction with the benzyl ring, the methyl group, and the pyran ring of the α-chromene group; the methyl group of the α-chromene group also engages in alkyl interactions with His438 and Ile442. The linear isopentenyl engages in a π–σ interaction with Tyr332 and π–alkyl or alkyl interactions with Ala328 and Phe329. The hydroxyl on the benzofuran ring interacts with Asn68 via a conventional hydrogen bond and with Ile69 via a carbon hydrogen bond. Additionally, the benzofuran ring engages in a π–anion interaction with Asp70 and forms a π–donor hydrogen bond with Thr120. 

**Figure 6:**
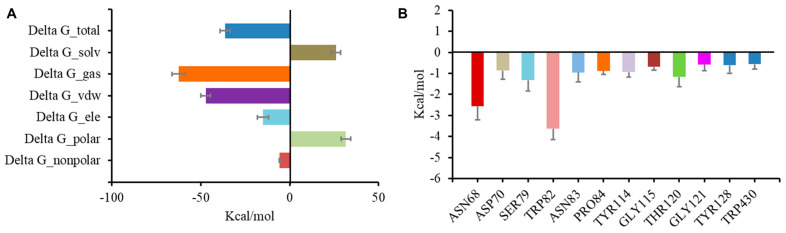
Molecular dynamic simulation results regarding the binding of BChE to compound **14**. Total binding free energy and its component for **14** with 5k5e (**A**); residue contribution to receptor–ligand complexes for 5k5e with **14** (**B**).

To evaluate the stability of the complex of BChE and the inhibitors, a molecular dynamics simulation was conducted in AMBER following preliminary docking. Cathafuran C (**14**) combined with BChE reached a steady state in 20 ns. Calculations using the MMGBSA method revealed a total binding free energy of −36.3 kcal/mol for the combination of **14** and BChE (Figure 6A). Among the binding free energies, van der Waals energy (ΔG_vdw, −47.25 kcal/mol) was the most important component, and electrostatic energy (ΔG_ele, −15.06 kcal/mol) also made a very positive contribution to EI binding. It was reported that π–π interactions of Trp82, Trp231, and Phe329 with the inhibitor and a hydrogen bond between His438 and the inhibitor were significant for inhibiting BChE [37,38,39]. The contributions of hot residues in the binding pocket of BChE were analyzed to identify the key residues in BChE for binding **14**. The residues with interaction energies lower than −1 kcal/mol are considered essential for ligand recognition and complexing. As shown in Figure 6B, Trp82 (−3.63 kcal/mol), Asn68 (−2.56 kcal/mol), Ser79 (−1.32 kcal/mol), and Thr120 (−1.16 kcal/mol) are regarded as key residues for the binding of compound **14** to BChE. Consistent with the molecular docking results, the π–π interaction of Trp82 with **14** and the hydrogen bond between Asn68 and **14** contributed substantially to the combination of BChE and **14**.

## 3. Materials and Methods

### 3.1. General Experimental Procedure

AB-8 macroporous resin (Tianjin Yunkai Resin Technology Co., Ltd., Tianjin, China), silica gel (200–300 mesh, Qingdao Marine Chemical Inc., Qingdao, China), polyamide (60–100 mesh, Taizhou Luqiao Sijia Biochemical Plastic Factory, Taizhou, China), and YMC*GEL^®^ ODS-A-HG (12 nm S-50 μm, YMC Co., Ltd., Kyoto, Japan) were used for column chromatography (CC). CC fractions were analyzed on an Agilent 1100 HPLC system equipped with a photo diode array detector (G1316A) using an analytical Kromasil C-18 column (5 μm, 100 Å, 4.6 mm × 250 mm; Akzo Nobel, Amsterdam, The Netherlands). Preparative HPLC was performed using a QuikSep chromatographic system (H&E, Beijing, China), and a Gemini C-18 column (21.2 mm × 250 mm, column temperature: 26 °C) was used for separation and purification. Optical rotations were measured using a P-2000 digital polarimeter (JASCO, Tokyo, Japan). UV spectra were recorded using a UV-2600 spectrophotometer (Shimadzu, Kyoto, Japan). HR-ESI-MS was measured using Xevo G2-XS QTOF mass spectrometer (Agilent, Santa Clara, CA, USA), and the NMR spectra were collected using a Bruker-500 spectrometer (Bruker, Karlsruhe, Germany) (500 MHz ^1^H and 125 MHz ^13^C-NMR). Circular dichroism (CD) tests were carried out using a Chirascan circular dichroism spectrometer (Applied Photophysics, Surrey, UK). A SynergyHTX micro plate reader (BioTek, Winooski, VT, USA) was used to read the absorbance in the enzymatic tests. 

AChE (EC 3.1.1.7, from electric eel), BChE (EC 3.1.1.8, from equine serum), acetylthiocholine iodide (ATCI), butyrylthiocholine iodide (BTCI), and 5,5′-dithiobis (2-nitrobenzoic acid) (DTNB) were purchased from Aladdin Industrial Co., Ltd. (Shanghai, China).

### 3.2. Plant Material

Cortex Mori Radicis was bought from Bozhou herb market, Bozhou, Anhui, China; collected from Anhui Province, China, in 2020; and identified by Professor Jing Hu (College of Traditional Chinese Medicine, Tianjin University of Traditional Chinese Medicine, Tianjin, China). A voucher specimen (TM-2003) is deposited in the College of Life Sciences and Agronomy, Zhoukou Normal University.

### 3.3. Extraction and Isolation

The Cortex Mori Radicis sample (9.3 kg) was extracted twice with 80% ethanol (30 L) at 80 °C. After filtration, the extraction solution was condensed in vacuo at 60 °C to a 10% ethanol suspension (approximately 20 L, with solid content of approximately 990 g). The suspension was subjected to an AB-8 macroporous adsorption resin column with a column volume (CV) of 4 L and eluted with gradients of 10%, 30%, 50%, 60%, 80%, and 95% ethanol (E) each 3 CVs. The 10% ethanol eluate was labeled as Fr-A (580 g), the 30% ethanol eluate was labeled as Fr-B (61.2 g), the 50~80% ethanol eluate was merged and labeled as Fr-C (180 g), and the 95% ethanol eluate was labeled Fr-D (50.8 g). Fr-C (180 g) was subjected to silica gel CC, and gradient elution was performed using dichloromethane (D)-methanol (M) (100:0–70:30) as an eluent to afford Fr-C1–Fr-C4.

Fr-C2 (7.0 g, eluted with DM 99:1–96:4) was subjected to ODS CC eluted with gradient aqueous methanol to obtain subfractions Fr-C21–Fr-C28. Fr-C23 (40%M eluate) was separated using preparative HPLC with 48% methanol as the mobile phase to afford compound **7** (13.4 mg, *t*_R_ = 67.3 min). The Fr-C24 (60%M eluate) was subjected to preparative HPLC (56%M, 10 mL/min) to obtain compounds **2** (23 mg, *t*_R_ = 19.0 min), **6** (65 mg, *t*_R_ = 42.8 min), and **1** (57 mg, *t*_R_ = 45.6 min). Fr-C26 (80%M eluate) was purified using preparative HPLC (65%M, 10 mL/min) to yield compounds **14** (56 mg, *t*_R_ = 28 min) and **16** (43 mg, *t*_R_ = 37 min). 

Fr-C3 (93 g, eluted with DM 92:8–90:10) was subjected to polyamide CC via gradient elution with 40–90%E to obtain subtractions Fr-C31–Fr-C36. Fr-C31 (40%E eluate) was further separated using silica gel CC to afford subfractions Fr-31-1–Fr-31-5. Fr-31-2 (DM 98:2) was performed using HPLC separation (64%M, 10 mL/min), affording compounds **3** (36 mg, *t*_R_ = 14.7 min) and **4** (45 mg, *t*_R_ = 19.8 min), and Fr-31-3 (DM 96:4) was separated using HPLC (50%M, 10 mL/min) to yield **5** (12 mg, *t*_R_ = 38.8 min). Fr-C33 (70%E eluate) was separated using silica gel CC to obtain subfractions Fr-C331–Fr-C-334; Fr-C331 was separated using ODS CC and MPLC and subsequently purified using preparative HPLC to obtain **8** (39 mg, *t*_R_ = 29 min, 60%M, 10 mL/min), **11** (35 mg, *t*_R_ = 33 min, 65%M, 10 mL/min), **15** (48 mg, *t*_R_ = 12 min, 75%M, 10 mL/min), and **9** (32 mg, *t*_R_ = 13 min, 75%M, 10 mL/min). Fr-C332 was subjected to preparative HPLC with 70%M as the mobile phase (10 mL/min) to yield compounds **10** (65 mg, *t*_R_ = 7.8 min), **13** (29 mg, *t*_R_ = 11.5 min), and **12** (23 mg, *t*_R_ = 10.5 min). 

Albaphenol A (**1**): yellow needle crystal (MeOH), m.p. 176–178 °C. UV (MeOH) λmax (logε): 203 (4.62), 270 (2.14), 310 (2.32), and 384 (4.47) nm. Negative HR-ESI-MS: *m*/*z* measured 215.0703 [M-H]^−^ (calculated for C_13_H_11_O_3_ [M-H]^−^ 215.0708). For ^1^H and ^13^C NMR data, see Table 1 and Table 2.

Albaphenol B (**2**): yellow amorphous powder. UV (MeOH) λmax (logε): 203 (4.54), 268 (2.31), 300 (2.09), 378 (4.38) nm. Positive HR-ESI-MS: *m*/*z* measured 249.1124 [M+H]^+^ (calculated for C_14_H_17_O_4_ [M+H]^+^ 249.1127). For ^1^H and ^13^C NMR data, see Table 1 and Table 2.

Albaphenol C (**3**): white amorphous powder, ${\left[\alpha \right]}_{D}^{24}$ −42.0°. UV (MeOH) λmax (logε): 282 (2.02) nm. Negative HR-ESI-MS: *m*/*z* measured 249.1126 [M-H]^−^ (calculated for C_14_H_17_O_4_ [M-H]^−^ 249.1127). For ^1^H and ^13^C NMR data, see Table 1 and Table 2.

Albaphenol D (**4**): white amorphous powder, ${\left[\alpha \right]}_{D}^{24}$ −15.3°. UV (MeOH) λmax (logε): 282 (2.05) nm. Negative HR-ESI-MS: *m*/*z* measured 263.1282 [M-H]^−^ (calculated for C_15_H_19_O_4_ [M-H]^−^ 263.1283). For ^1^H and ^13^C NMR data, see Table 1 and Table 2.

Albaphenol E (**5**): white amorphous powder. UV (MeOH) λmax (logε): 280 (2.15) nm. Negative HR-ESI-MS: *m*/*z* measured 223.0966 [M-H]^−^ (calculated for C_12_H_15_O_4_ [M-H]^−^ 223.0970). For ^1^H and ^13^C NMR data, see Table 1 and Table 2. 

Crystal data for **1**: C_13_H_12_O_3_, *M* = 216.23; space group: *P*-1, *Z* = 4; cell: *a* = 10.088 Å, *b* = 10.186 Å, *c* = 11.621 Å, *α* = 73.743(4)°, *β* = 81.695(3)°, *γ* = 81.015(4)°, *T* = 293 K, *μ*(MuKα) = 0.742 mm^−1^, *h* = 12, *k* = 12, *l*max = 13, *N*ref = 4018, *T*min = 0.945, *T*max = 1.000, Theta (max) = 67.069. *R* (reflections) = 0.0541 (2513), *wR*^2^ (reflections) = 0.1701 (4018), *S* = 1.043, *N*par = 298. Crystal data for **6**: C_13_H_12_O_3_, *M* = 216.23; space group: *P*-1, *Z* = 4; cell: *a* = 7.198 Å, *b* = 17.49 Å, *c* = 18.505 Å, *α* = 97.983 (2)°, *β* = 93.2845 (18)°, *γ* = 92.216(2)°, *T* = 293 K, *μ*(MuKα) = 0.779 mm^−1^, *h* = 8, *k* = 20, *l*max = 22, *N*ref = 8224, *T*min = 0.845, *T*max = 1.000, Theta (max) = 67.080. *R* (reflections) = 0.0598 (4933), *wR*^2^ (reflections) = 0.1966 (8210), *S* = 1.022, *N*par = 619. The crystallographic data for **1** (CCDC 2267221) and **6** (CCDC 2267218) have been deposited at the Cambridge Crystallographic Data Center (CCDC). 

### 3.4. ChE Inhibitory Activity Assay

The AChE (EC 3.1.1.7) and BChE (EC 3.1.1.8)-inhibitory activities of compounds **1**–**17** were determined following Ellman’s method [40] with some modifications, referring to our previously published work [15]. Briefly, AChE and BChE solutions (0.2 units/mL) and ATCI, BTCI, and DTNB solutions (10 mM) were prepared in PBS solution (0.1 M, pH 8.0). The test compound stock solutions (10 mM) were made using methanol, and five different concentrations of each compound were prepared by doubling the dilution with methanol to determine the half-maximal inhibitory concentration (IC_50_). Firstly, 160 μL of PBS, 2 μL of the test samples, 20 μL of AChE or BChE, and 10 μL of DTNB were mixed and preincubated at 37 °C for 10 min. Thereafter, 10 μL of ATCI or BTCI was added to initiate the reaction, which was incubated at 37 °C for 25 min. The absorbance was measured at 412 nm during incubation. The inhibition rate was calculated using the following formula: IR% = [(A_c_ − A_s_)]/(A_c_ − A_b_] × 100%, where A_b_ denotes the absorbance of the blank control (20 μL of water replacing the enzyme solution), A_c_ represents the absorbance of the control (2 μL of methanol replacing the sample solutions), and A_s_ denotes the absorbance of the sample. All of the tests for each sample were performed in triplicate. The IC_50_ value of each compound was obtained by plotting the inhibition rate against the logarithm of its concentration, and the data were expressed as the mean ± SD. 

### 3.5. Kinetic Study of BChE Inhibition

Using the same protocol as the inhibitory activity assay, kinetic studies of BChE inhibition were performed with a series of concentrations of substrate BTCI, namely, 0.1, 0.2, 0.3, 0.4, and 0.5 mM or 0.2, 0.4, 0.5, 0.6, and 0.7 mM. Three concentrations of each test compound were selected according to the IC_50_ value. The detection of absorbance was conducted during the enzymatic reaction at 10 min, 20 min, and 25 min. The change in absorbance per minute was referred to as the velocity of the enzyme-promoting reaction. The Lineweaver–Burk plots (double-reciprocal plots) were created by plotting the reciprocal of velocity (1/V) against the reciprocal of BTCI concentration (1/[BTCI]) for a compound at three concentrations, and the kinetic parameters, Michaelis constant (Km), and maximum velocity (V_max_) obtained therefrom were used to check the inhibition modes. The value of the inhibitor constant Ki was determined by referencing the secondary plot of the slope of the double-reciprocal lines (from the Lineweaver–Burk plot) as a function of [I], and the x intercept was equal to −Ki. Another secondary plot was fitted by 1/V_max_ as a function of the inhibitor concentration [I], and the value of −αKi can be determined to be the x intercept therefrom [35,36].

### 3.6. Molecular Docking and Molecular Dynamics Studies

The binding modes of BChE–inhibitors complex were investigated via docking calculations, which were performed using Autodock Vina software in Yinfo Cloud Platform (http://cloud.yinfotek.com/) [41]. The detailed procedure was in accordance with what we published previously [42]. In brief, 3D structures of compounds were generated and then energetically minimized with MM_2_ force field to a minimum root mean square (RMS) gradient of 0.005 using Chem3D Ultra 2017 (Version 17.0.0.206). The crystal structures of BChE (PDB code: 5k5e [43]) were extracted from the Protein Data Bank and further prepared by removing water, ions, and original ligands. Subsequently, the grid boxes were prepared using AutoGrid. For BChE, the dimensions of the grid were set to 26 × 26 × 26, and the grid box center was situated at coordinates of x = 2.967, y = 4.171, and z = 9.571, corresponding to Trp82 residue. All of the parameters were set as default for the simulated annealing. The accomplished docking procedure afforded nine top-ranked ligand–receptor conformations sorted by the calculated free energy of binding. The best pose of each ligand with the highest affinity score (kcal/mol) was visualized using Discovery Studio Visualizer v21.1.0.20298 (Accelrys, San Diego, CA, USA) for analyzing the interaction modes between the enzyme and inhibitors. 

Following preliminary docking, the PMEMD module in AMBER 20 was used to determine the molecular dynamics (MD) of the combination between the enzyme and inhibitor. Firstly, an AMBER ff99SB forcefield and the general AMBER forcefield were applied for the structural preparation of protein and ligand, respectively [44]. The simulation systems were solvated in a TIP3P water box in a 10 Å hexahedron and were neutralized by adding sodium ions. To reduce possible steric stresses, the simulation procedure was started with two steps of minimization each set to 1000 steps. Then, the systems were heated to 300 K using a Langevin thermostat over 20 ps in linear way, which was under an NVT ensemble with weak restraints of 10 kcal/mol/Å^2^ on the protein backbone atoms. Thereafter, under 1 atm and 300 K conditions, a step of equilibration under NPT ensemble was set using Langevin thermostat over 200 ps, followed by another equilibration under NVT ensemble using Berendson thermostat over 1 ns. Finally, frames were extracted from 20 ns of the trajectory for CPPTRAJ analysis [45]. The MMGBSA method was applied in AMBER 20 to calculate the binding free energy and its decomposition [46]. 

## 4. Conclusions

In summary, five novel compounds, including a new prenylated benzofuranone (**1**) and four ring-opening derivatives (**2**–**5**), as well as nigranol A (**6**), together with ten benzofuran-type stilbenes (**7**–**16**), were isolated from Cortex Mori Radicis and identified using spectroscopic methods (1D and 2D NMR and HR-ESI-MS). Albaphenol A (1) showed a moderate inhibitory effect on BChE (IC_50_ 51.0 μM) in a mixed uncompetitive manner, while its trans isomer (**6**) and ring-opening derivatives showed no obvious inhibition of AChE or BChE. The configurations of the Δ_7,8_-double bonds for **1** and **6** were determined unambiguously using XRD analysis, and the structure of nigranol A (**6**) was revised therefrom. The ten 2-arylbenzofuran derivatives (benzofuran-type stilbenes), with or without prenylation at different positions, were also evaluated for their ChEs inhibitory activities. Among them, six prenylated ones showed significant inhibitory activity, and the linear-type prenyl or the cyclization of prenyl that transformed it into an α-chromene group was essential for BChE inhibition, which is consistent with previous studies suggesting that prenyl and geranyl groups play important roles in enzyme inhibition [15,20,42]. For the first time, cathafuran C (**14**) was discovered to possess potent and selective inhibitory activity to BChE in a competitive manner (Ki = 1.7 μM). Molecular docking suggested that **14** could properly insert itself into the catalytic pocket, forming several kinds of intermolecular interactions with different amino acid residues. Further molecular dynamic simulations showed that **14** could engage in π–π interactions with Trp82 and form a hydrogen bond with Asn68, which substantially contributed to the combination and inhibition of BChE. This study provides a further theoretical foundation for using Cortex Mori Radicis and its constituents as functional agents for AD treatment. However, there are few in vivo studies evaluating the anti-AD activity of Cortex Mori Radicis or its representative constituents. Therefore, further in vivo studies should be performed to research the anti-AD function and mechanism of the bioactive NPs from Cortex Mori Radicis.

## Figures and Tables

**Table 1:** ^1^H NMR data of compounds **1**–**6** (500 MHz, δ in ppm, *J* in Hz).

No.	1 ^a^	2 ^a^	3 ^b^	4 ^b^	5 ^b^	6 ^a^
2	6.59 d (2.2)	6.42 d (2.4)	6.26 d (2.4)	6.28 d (2.4)	6.25 d (2.4)	6.63 d (2.2)
4	6.65 dd (8.3, 2.2)	6.37 dd (8.2, 2.4)	6.23 dd (8.3, 2.4)	6.24 dd (8.3, 2.4)	6.37 dd (8.8, 2.4)	6.68 dd (8.4, 2.2)
5	7.54 d (8.3)	6.80 d (8.2)	6.91 d (8.3)	6.93 d (8.3)	7.77 d (8.8)	7.67 d (8.4)
7	—	—	3.85 dd (8.0, 7.3)	3.83 dd (8.2, 7.2)	—	—
8	7.64 d (12.2)	7.60 d (11.8)	Ha 2.59 ddd (14.6, 8.0, 7.3) Hb 2.37 dt (14.6, 7.3)	Ha 2.59 ddd (14.7, 8.2, 7.2) Hb 2.36 dt (14.7, 7.2)	3.10–2.93 m	7.36 d (12.6)
9	7.39 br d (12.2)	5.86 br d (11.8)	5.08 br t (7.3)	5.09 br t (7.2)	1.96–1.76 m	6.81 br d (12.6)
11	2.04 br s	1.91 br s	1.64 d (1.4)	1.63 d (1.7)	1.24 s	2.04 br s
12	2.01 br s	1.79 br s	1.57 d (1.3)	1.57 d (1.4)	1.24 s	2.07 br s
1′	—	3.64 s	3.62 s	4.09 dq (16.6, 7.1) 4.07 dq (16.6, 7.1)	—	—
2′	—	—	—	1.18 t (7.1)	—	—
1-OH	—	7.97 br s	—	—	—	—
3-OH	8.93 br s	8.26 br s	—	—	—	8.94 br s

^a^ Tested in acetone-*d*_6_, and chemical shift values were recorded using the solvent signal at δ_H_ 2.05 as reference. ^b^ Tested in methanol-*d*_4_, and chemical shift values were recorded using the solvent signal at δ_H_ 3.31 as reference. — No signal.

**Table 2:** ^13^C NMR data of compounds **1**–**6** (125 MHz, δ in ppm) ^a^.

No.	1 ^b^	2 ^b^	3 ^c^	4 ^c^	5 ^c^	6 ^b^
1	154.9 s	157.0 s	157.0 s	156.9 s	166.4 s	155.9 s
2	98.9 d	103.6 d	103.4 d	103.4 d	103.7 d	99.2 d
3	160.0 s	159.2 s	158.3 s	158.2 s	166.5 s	160.1 s
4	111.9 d	107.3 d	107.5 d	107.4 d	109.1 d	112.1 d
5	121.9 d	133.1 d	129.9 d	129.8 d	133.7 d	125.4 d
6	117.5 s	115.2 s	118.3 s	118.4 s	113.9 s	115.9 s
7	117.9 s	128.4 s	45.6 d	45.8 d	206.5 s	118.7 s
8	133.2 d	136.8 d	32.1 t	32.1 t	34.0 t	132.2 d
9	122.3 d	123.2 d	123.0 d	122.9 d	39.1 t	122.0 d
10	151.0 s	145.3 s	134.2 s	134.1 s	70.9 s	153.0 s
11	18.7 q	18.9 q	25.9 q	25.9 q	29.2 q	18.9 q
12	27.3 q	26.8 q	17.8 q	17.8 q	29.2 q	27.2 q
13	167.8 s	169.1 s	177.3 s	176.9 s	—	170.1 s
1′	—	51.8 q	52.1 q	61.5 t	—	—
2′	—	—	—	14.5 q	—	—

^a^ Signals assignments were based on the results of DEPT, HMQC and HMBC experiments. Multiplicities of the carbon signals were determined by DEPT experiments and are indicated by s (singlet), d (doublet), t (triplet) and q (quartet). ^b^ Tested in acetone-*d*_6_, and chemical shift values were recorded using the solvent signal at δ_C_ 29.84 as reference. ^c^ Tested in methanol-*d*_4_, and chemical shift values were recorded using the solvent signal at δ_C_ 49.00 as reference. — No signal.

**Table 3:** The inhibitory activities against ChEs of compounds **1**–**16**.

Compounds	IC_50_ μM ^a^		BChE			
	AChE	BChE	Ki μM	αKi μM	α	Inhibition Type
**1**	>100	45.5 ± 5.9	93.4	43.0	0.46	Mixed uncompetitive
**2**	>100	>100	—	—	—	—
**3**	>100	>100	—	—	—	—
**4**	>100	61.0 ± 3.4	—	—	—	—
**5**	>100	>100	—	—	—	—
**6**	>100	>100	—	—	—	—
**7**	>100	>100	—	—	—	—
**8**	>100	27.9 ± 3.5	12.8	71.1	5.55	Mixed competitive
**9**	81.2 ± 3.9	9.5 ± 3.2	5.7	25.2	4.42	Mixed competitive
**10**	>100	>100	—	—	—	—
**11**	40.5 ± 4.7	13.5 ± 1.6	12.7	13.3	1.05	Noncompetitive
**14**	>100	2.6 ± 0.2	1.7	22.7	13.35	Competitive
**15**	>100	27.1 ± 6.8	—	—	—	—
**16**	>100	32.8 ± 4.2	—	—	—	—
Galantamine ^b^	0.8 ± 0.2	35.3 ± 5.6	—	—	—	—

^a^ Sample concentration that led to 50% enzyme activity loss. ^b^ Galantamine used as positive control. — Not tested.

## Data Availability

Data are contained within the article and supplementary materials. The crystallographic data for **1** (CCDC 2267221) and **6** (CCDC 2267218) can be obtained, free of charge, upon application to CCDC, 12 Union Road, CB2 1EZ, UK (Fax: +44-0-1223-336033 or e-mail: deposit@ccdc.cam.ac.uk).

## Supplementary Materials

The following supporting information can be downloaded at https://www.mdpi.com/article/10.3390/molecules29020315/s1. Figure S1: HPLC detection of cis-trans-tautomerism of compounds **1** and **6**; Figure S2. Chiral HPLC analysis of compound **4**; Figure S3: Secondary plots for the determination of the inhibitor constants; Figures S4–S44: HRESIMS, ^1^H NMR, ^13^C NMR, ^1^H-^1^H COSY, HSQC, HMBC, and ROESY spectra of compounds **1**–**6**, and **14**.

## References

[1] Alzheimer’s Disease International (2019). World Alzheimer Report 2019: Attitudes to Dementia [R].

[2] Goedert M., Spillantini M.G. (2006). A century of Alzheimer’s disease. Science.

[3] Grundke-Iqbal I., Iqbal K., Tung Y.C., Quinlan M., Wisniewski H.M., and Binder L.I. (1986). Abnormal phosphorylation of the microtubule associated protein tau (tau) in Alzheimer cytoskeletal pathology. Proc. Natl. Acad. Sci. USA.

[4] Linker R.A., Lee D.H., Ryan S., van Dam A.M., Conrad R., Bista P., Zeng W., Hronowsky X., Buko A., Chollate S. (2011). Fumaric acid esters exert neuroprotective effects in neuroinflammation via activation of the Nrf2 antioxidant pathway. Brain.

[5] Aliev G., Priyadarshini M., Reddy V.P., Grieg N.H., Kaminsky Y., Cacabelos R., Ashraf G.M., Jabir N.R., Kamal M.A., Nikolenko V.N. (2014). Oxidative stress mediated mitochondrial and vascular lesions as markers in the pathogenesis of Alzheimer disease. Curr. Med. Chem..

[6] Craig L.A., Hong N.S., McDonald R.J. (2011). Revisiting the cholinergic hypothesis in the development of Alzheimer’s disease. Neurosci. Biobehav. Rev..

[7] Li Q., He S., Chen Y., Feng F., Qu W., Sun H. (2018). Donepezil-based multi-functional cholinesterase inhibitors for treatment of Alzheimer’s disease. Eur. J. Med. Chem..

[8] Li Q., Chen Y., Xing S., Liao Q., Xiong B., Wang Y., Lu W., He S., Feng F., Liu W. (2021). Highly potent and selective butyrylcholinesterase inhibitors for cognitive improvement and neuroprotection. J. Med. Chem..

[9] Rossi M., Freschi M., de Camargo Nascente L., Salerno A., de Melo Viana Teixeira S., Nachon F., Chantegreil F., Soukup O., Prchal L., Malaguti M. (2021). Sustainable drug discovery of multi-target-directed ligands for Alzheimer’s Disease. J. Med. Chem..

[10] Xing S., Sun H. (2023). The role of butyrylcholinesterase in Alzheimer’s disease and design strategies of its inhibitors. Prog. Pharm. Sci..

[11] Orhan I.E., Senol F.S., Shekfeh S., Skalicka-Wozniak K., Banoglu E. (2017). Pteryxin−A promising butyrylcholinesterase-inhibiting coumarin derivative from Mutellina purpurea. Food Chem. Toxicol..

[12] Jabir N.R., Khan F.R., and Tabrez S. (2018). Cholinesterase targeting by polyphenols: A therapeutic approach for the treatment of Alzheimer’s disease. CNS Neurosci. Ther..

[13] Wu C., Tu Y.B., Li Z., Li Y.F. (2019). Highly selective carbamate-based butyrylcholinesterase inhibitors derived from a naturally occurring pyranoisoflavone. Bioorganic Chem..

[14] Chen X., Drew J., Berney W., Lei W. (2021). Neuroprotective natural products for Alzheimer’s disease. Cells.

[15] Cui X., Deng S., Li G., Zhang Y., Wang L., Wu C., Deng Y. (2022). Butenolide derivatives from Aspergillus terreus selectively inhibit butyrylcholinesterase. Front. Chem..

[16] The State Pharmacopoeia Commission of P. R. (2020). China. Pharmacopoeia of People’s Republic of China.

[17] Chan E.W.C., Lye P.Y., Wong S.K. (2016). Phytochemistry, pharmacology, and clinical trials of Morus alba. Chin. J. Nat. Med..

[18] Wei H., Zhu J.J., Liu X.Q., Feng W.H., Wang Z.M., Yan L.H. (2016). Review of bioactive compounds from root barks of Morus plants (Sang-Bai-Pi) and their pharmacological effects. Cogent Chem..

[19] Paudel P., Yu T., Seong S.H., Kuk E.B., Jung H.A., Choi J.S. (2018). Protein tyrosine phosphatase 1B inhibition and glucose uptake potentials of mulberrofuran G, albanol B, and kuwanon G from root bark of Morus alba L. in insulin-resistant HepG2 cells: An in vitro and in silico study. Int. J. Mol. Sci..

[20] Seong S.H., Ha M.T., Min B.S., Jung H.A., Choi J.S. (2018). Moracin derivatives from Morus Radix as dual BACE1 and cholinesterase inhibitors with antioxidant and anti-glycation capacities. Life Sci..

[21] Paudel P., Seong S.H., Zhou Y., Ha M.T., Min B.S., Jung H.A., Choi J.S. (2019). Arylbenzofurans from the Root Bark of Morus alba as Triple Inhibitors of Cholinesterase, β-Site Amyloid Precursor Protein Cleaving Enzyme 1, and Glycogen Synthase Kinase-3β: Relevance to Alzheimer’s Disease. ACS Omega.

[22] Kuk E.B., Jo A.R., Oh S.I., Sohn H.S., Seong S.H., Roy A., Choi J.S., Jung H.A. (2017). Anti-Alzheimer’s disease activity of compounds from the root bark of *Morus alba* L.. Arch. Pharmacal Res..

[23] Xia C.L., Tang G.H., Guo Y.Q., Xu Y.K., Huang Z.S., Yin S. (2019). Mulberry Diels-Alder-type adducts from Morus alba as multi-targeted agents for Alzheimer’s disease. Phytochemistry.

[24] Xu L., Yu M., Niu L., Huang C., Wang Y., Wu C., Yang P., Hu X. (2020). Phenolic compounds isolated from Morus nigra and their α-glucosidase inhibitory activities. Nat. Prod. Res..

[25] Takasugi M., Nagao S., Masamune T., Shirata A., Takahashi K. (1978). Structure of moracin A and B, new phytoalexins from diseased mulberry. Tetrahedron Lett..

[26] Takasugi M., Nagao S., Ueno S., Masamune T., Shirata A., Takahashi K. (1978). Moracin C and D, new phytoalexins from diseased mulberry. Chem. Lett..

[27] Zhou C.X., Tanaka J., Cheng C.H., Higa T., Tan R.X. (1999). Steroidal alkaloids and stilbenoids from *Veratrum taliense*. Planta Medica.

[28] Jeong S.H., Ryu Y.B., Curtis-Long M.J., Ryu H.W., Baek Y.S., Kang J.E., Lee W.S., Park K.H. (2009). Tyrosinase inhibitory polyphenols from roots of *Morus lhou*. J. Agric. Food Chem..

[29] Kaur N., Xia Y., Jin Y., Dat N.T., Gajulapati K., Choi Y., Hong Y.S., Lee J.J., Lee K. (2009). The first total synthesis of moracin O and moracin P, and establishment of the absolute configuration of moracin O. Chem. Commun..

[30] Ni G., Zhang Q.J., Zheng Z.F., Chen R.Y., Yu D.Q. (2009). 2-Arylbenzofuran Derivatives from *Morus cathayana*. J. Nat. Prod..

[31] Nomura T., Fukai T., Pei Y.H., Xu C.Q., Chen Y.J. (1996). Components of the root bark of *Morus cathayana*. 1. Structures of five new isoprenylated flavonoids, sanggenols A-E and a diprenyl-2-arylbenzofuran, mulberrofuran V. Heterocycles.

[32] Hano Y., Kohno H., Itoh M., Nomura T. (1985). Structures of three new 2-arylbenzofuran derivatives from the chinese crude drug ‘sang-bai-pi’ (morus root bark). Chem. Pharm. Bull..

[33] Sakuma M., Sakakura A., Ishihara K. (2013). Kinetic resolution of racemic carboxylic acids through asymmetric protolactonization promoted by chiral phosphonous acid diester. Org. Lett..

[34] Wu T., Zhu J.X., Wei Q., Li P., Wang L.B., Huang J., Wang J.H., Tang L.K., Wu L.J., Li C. (2019). Preparative separation of four isomers of synthetic anisodamine by HPLC and diastereomer crystallization. Chirality.

[35] Copeland R.A. (2000). Enzymes: A Practical Introduction to Structure, Mechanism, and Data Analysis.

[36] Buker S.M., Boriack-Sjodin P.A., Copeland R.A. (2019). Enzyme-inhibitor interactions and a simple, rapid method for determining inhibition modality. SLAS Discov..

[37] Nicolet Y., Lockridge O., Masson P., Fontecilla-Camps J.C., Nachon F. (2003). Crystal structure of human butyrylcholinesterase and of its complexes with substrate and products. J. Biol. Chem..

[38] Nachon F., Carletti E., Ronco C., Trovaslet M., Nicolet Y., Jean L., Renard P.Y. (2013). Crystal structures of human cholinesterases in complex with huprine W and tacrine: Elements of specificity for anti-Alzheimer’s drugs targeting acetyl- and butyryl-cholinesterase. Biochem. J..

[39] Knez D., Brus B., Coquelle N., Sosic I., Sink R., Brazzolotto X., Mravljak J., Colletier J.P., Gobec S. (2015). Structure-based development of nitroxoline derivatives as potential multifunctional anti-Alzheimer agents. Bioorganic Med. Chem..

[40] Ellman G.L., Courtney K.D., Andres V.J., Feather-Stone R.M. (1961). A new and rapid colorimetric determination of acetylcholinesterase activity. Biochem. Pharmacol..

[41] Dighe S.N., Deora G.S., De la Mora E., Nachon F., Chan S., Parat M.O., Brazzolotto X., Ross B.P. (2016). Discovery and structure-activity relationships of a highly selective butyrylcholinesterase Inhibitor by structure-based virtual screening. J. Med. Chem..

[42] Wu C., Cui X., Sun L., Lu J., Li F., Song M., Zhang Y., Hao X., Tian C., Song M. (2021). Aspulvinones suppress postprandial hyperglycemia as potent α-glucosidase inhibitors from *Aspergillus terreus* ASM-1. Front. Chem..

[43] Trott O., Olson A.J. (2010). AutoDock Vina: Improving the speed and accuracy of docking with a new scoring function, efficient optimization, and multithreading. J. Comput. Chem..

[44] Hornak V., Abel R., Okur A., Strockbine B., Roitberg A., Simmerling C. (2006). Comparison of multiple Amber force fields and development of improved protein backbone parameters. Proteins.

[45] Roe D.R., Cheatham T.E. (2013). PTRAJ and CPPTRAJ: Software for processing and analysis of molecular dynamics trajectory data. J. Chem. Theory Comput..

[46] Ylilauri M., Pentikäinen O.T. (2013). MMGBSA as a tool to understand the binding affinities of filamin-peptide interactions. J. Chem. Inf. Model..

