# Supplementary material for: Benzofuran Derivatives from Cortex Mori Radicis and Their Cholinesterase-Inhibitory Activity
Source: Molecules. 2024 Jan 8;29(2):315. doi: 10.3390/molecules29020315 (PMC10820097; doi:10.3390/molecules29020315)
Supplement: Supplementary file 1 [file molecules-29-00315-s001.zip › molecules-2769279-supplementary.pdf]

Supplementary Materials

Benzofuran derivatives from Cortex Mori Radicis and their cho-linesterase inhibitory activity

Xiang Cui[1,2], Ze-Hong Huang[1], Shan-Shan Deng[1], Yun-Xia Zhang[1], Guo-Yin Li[1], Li-Ning Wang[2], Yan-Ru Deng[2]* and Chang-Jing Wu[1,3]*

[1] College of Life Sciences and Agronomy, Zhoukou Normal University, Zhoukou 466001, China
[2] College of Traditional Chinese Medicine, Tianjin University of Traditional Chinese Medicine, Tianjin 301617, China
[3] Field Observation and Research Station of Green Agriculture in Dancheng County, Zhoukou 466001, China
* Correspondence: dengyanru@tjutcm.edu.cn (Y.-R.D.); wucj2009@163.com (C.-J.W.)

List of Supplementary Materials

Figure S1. HPLC detection of cis-trans-tautomerism of compounds 1 and 6 1
Figure S2. Chiral HPLC analysis of compound 4.......... 1
Figure S3. Secondary plots for the determination of the inhibitor constants.......... 2
Figure S4. HRESIMS data of compound 1.......... 3
Figure S5. ^{1}H-NMR spectrum of compound 1 in acetone-d_6 3
Figure S6. ^{13}C-NMR and DEPT-135 spectra of compound 1 in acetone-d_6 4
Figure S7. HSQC spectrum of compound 1 4
Figure S8. ^{1}H-^{1}H COSY spectrum of compound 1 5
Figure S9. HMBC spectrum of compound 1 5
Figure S10. ROESY spectrum of compound 1 6
Figure S11. HRESIMS data of compound 2.......... 6
Figure S12. ^{1}H-NMR spectrum of compound 2 in acetone-d_6 7
Figure S13. ^{13}C-NMR and DEPT-135 spectra of compound 2 in acetone-d_6 7
Figure S14. HSQC spectrum of compound 2 8
Figure S15. ^{1}H-^{1}H COSY spectrum of compound 2 8
Figure S16. HMBC spectrum of compound 2 9
Figure S17. ROESY spectrum of compound 2 9
Figure S18. HRESIMS data of compound 3.......... 10
Figure S19. ^{1}H-NMR spectrum of compound 3 in CD_3OD 10
Figure S20. ^{13}C-NMR and DEPT-135 spectra of compound 3 in CD_3OD.......... 11
Figure S21. HSQC spectrum of compound 3 11
Figure S22. ^{1}H-^{1}H COSY spectrum of compound 3 12
Figure S23. HMBC spectrum of compound 3 12
Figure S24. CD spectrum of compound 3 13
Figure S25. HRESIMS data of compound 4.......... 13
Figure S26. ^{1}H-NMR spectrum of compound 4 in CD_3OD 14
Figure S27. ^{13}C-NMR and DEPT-135 spectra of compound 4 in CD_3OD.......... 14
Figure S28. HSQC spectrum of compound 4 15
Figure S29. ^{1}H-^{1}H COSY spectrum of compound 4 15
Figure S30. HMBC spectrum of compound 4 16
Figure S31. CD spectrum of compound 4 16
Figure S32. HRESIMS data of compound 5.......... 17
Figure S33. ^{1}H-NMR spectrum of compound 5 in CD_3OD 17
Figure S34. ^{13}C-NMR and DEPT-135 spectra of compound 5 in CD_3OD.......... 18
Figure S35. HSQC spectrum of compound 5 18
Figure S36. ^{1}H-^{1}H COSY spectrum of compound 5 19
Figure S37. HMBC spectrum of compound 5 19
Figure S38. ^{1}H-NMR spectrum of compound 6 in acectone-d_6.......... 20
Figure S39. ^{13}C-NMR and DEPT-135 spectra of compound 6 in acectone-d_6.......... 20
Figure S40. HSQC spectrum of compound 6 21
Figure S41. ^{1}H-^{1}H COSY spectrum of compound 6 21
Figure S42. HMBC spectrum of compound 6 22
Figure S43. ^{1}H-NMR spectrum of compound 14 in CD_3OD 22
Figure S44. ^{13}C-NMR spectrum of compound 14 in CD_3OD 23

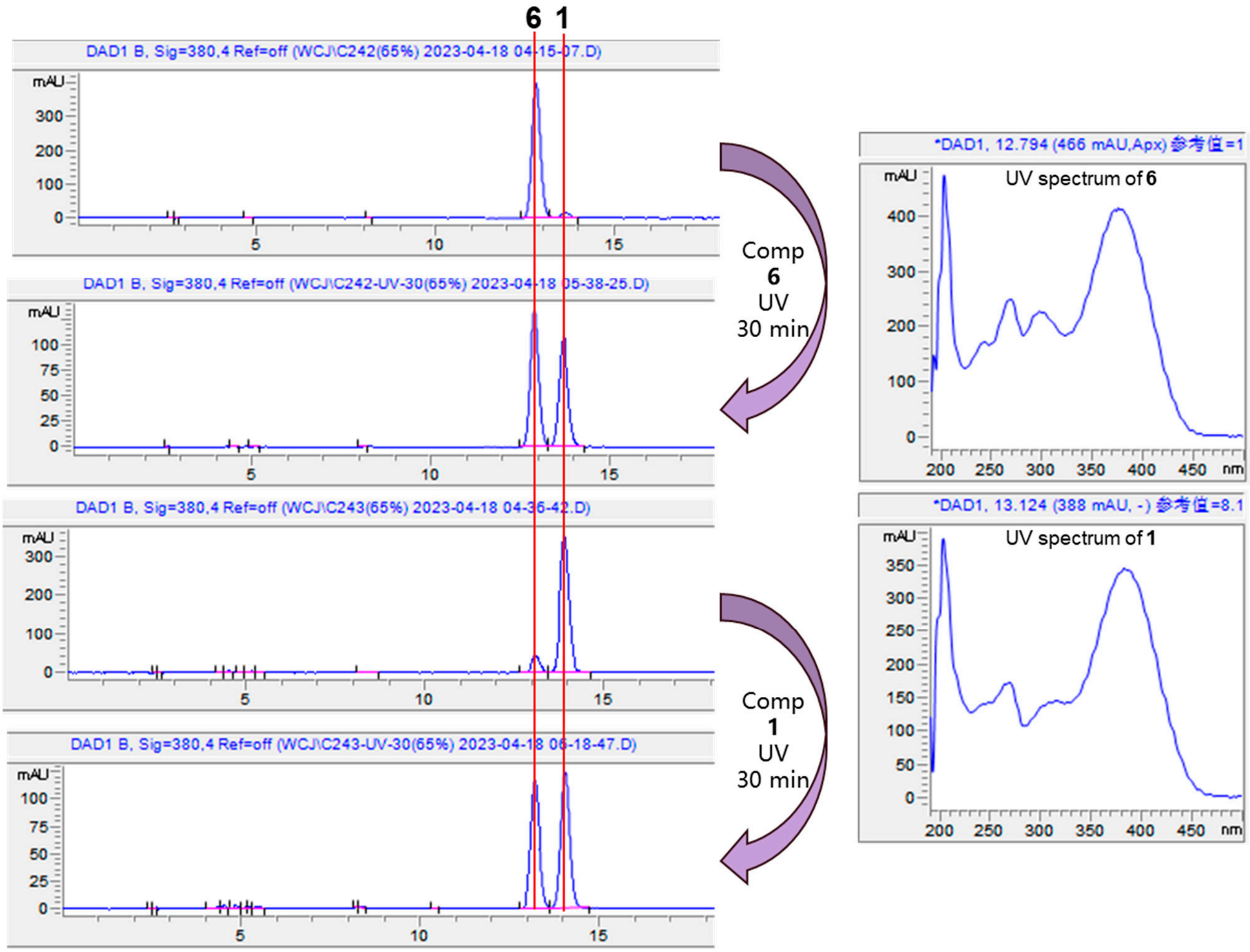

Figure S1. HPLC detection of cis-trans-tautomerism of compounds **1** and **6** promoted by UV exposure. Samples were analyzed on an Agilent 1100 HPLC system using an analytical Kromasil C-18 column (5 μm, 100 Å, 4.6 mm × 250 mm; Akzo Nobel), with 65% aqueous methanol as mobile phase, detected at 380 nm.

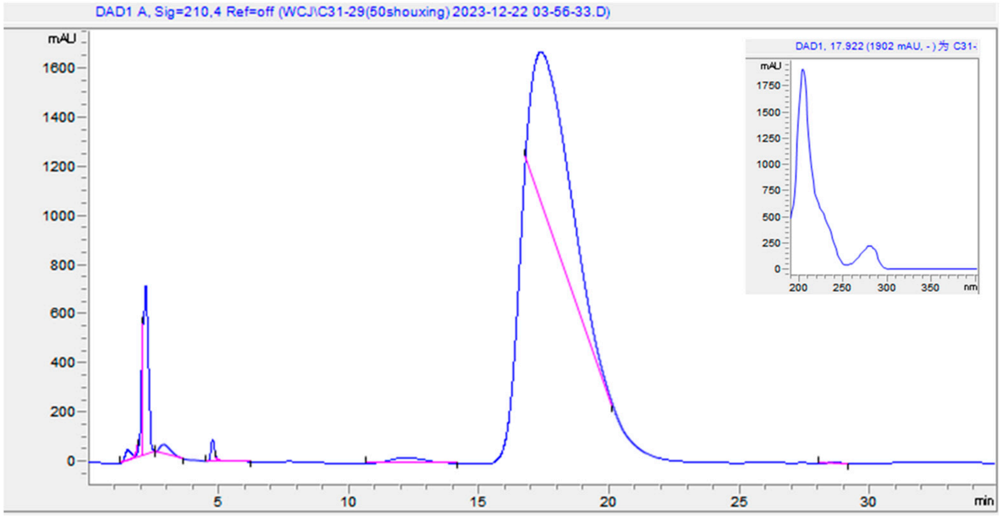

Figure S2. Chiral HPLC analysis of compound **4**. The HPLC analysis was conducted with a CHIRALPAK® AD-RH column (5 μm,4.6 mm*150 mm, Daicel Chiral Technologies (China) CO., LTD.) using 50% methanol as mobile phase with flow rate of 1ml/min at 30°C.

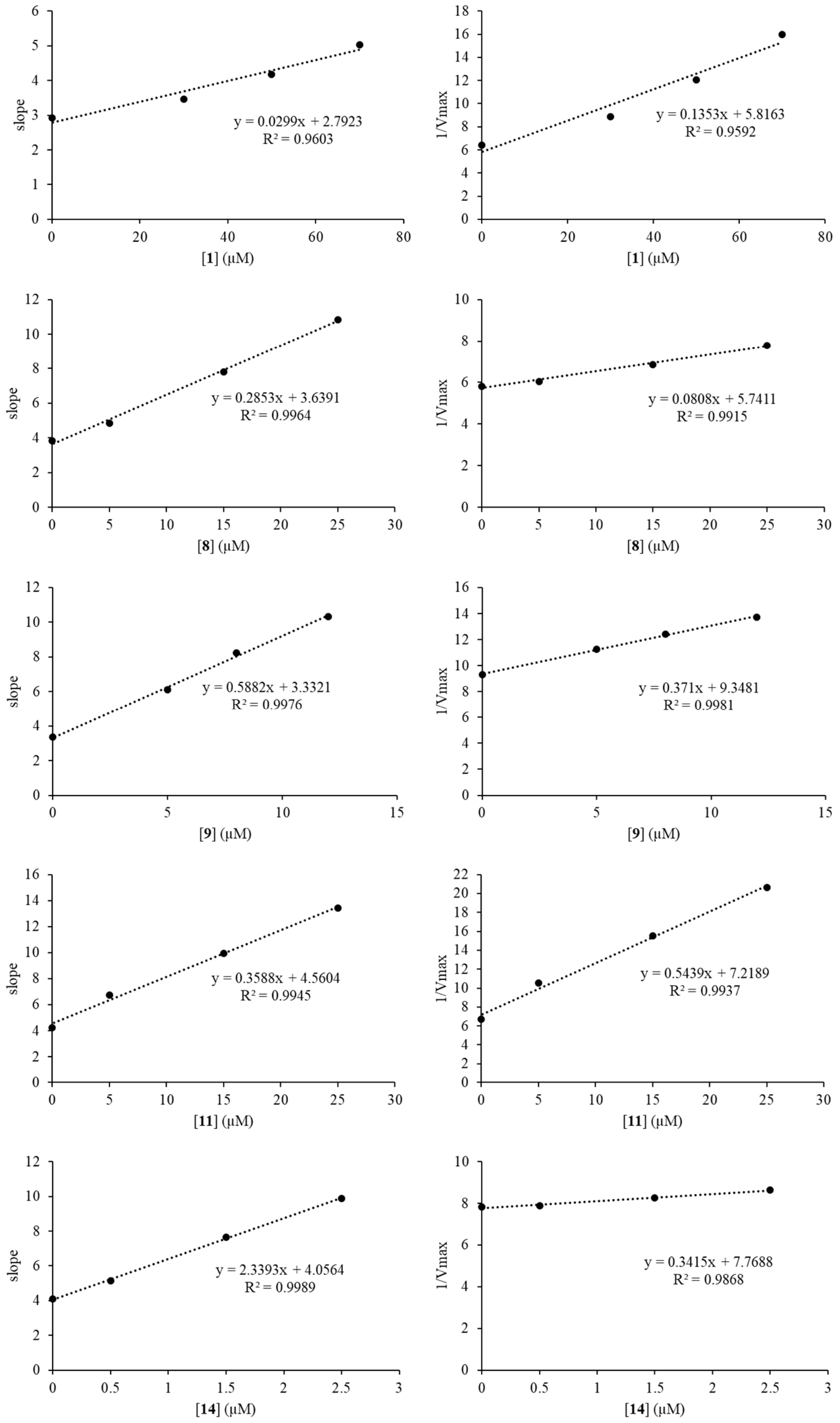

Figure S3. Secondary plots for the determination of the inhibitor constants for mixed-type inhibitors **1**, **8**, **9**, **11**, and **14**. 1/V is plotted as a function of [I], and the value of $-\alpha$Ki is determined from the x intercept of the line. The value of -Ki is determined from the x intercept of a plot of the slope of the lines from the double-reciprocal (Lineweaver— Burk) plot as a function of [I].

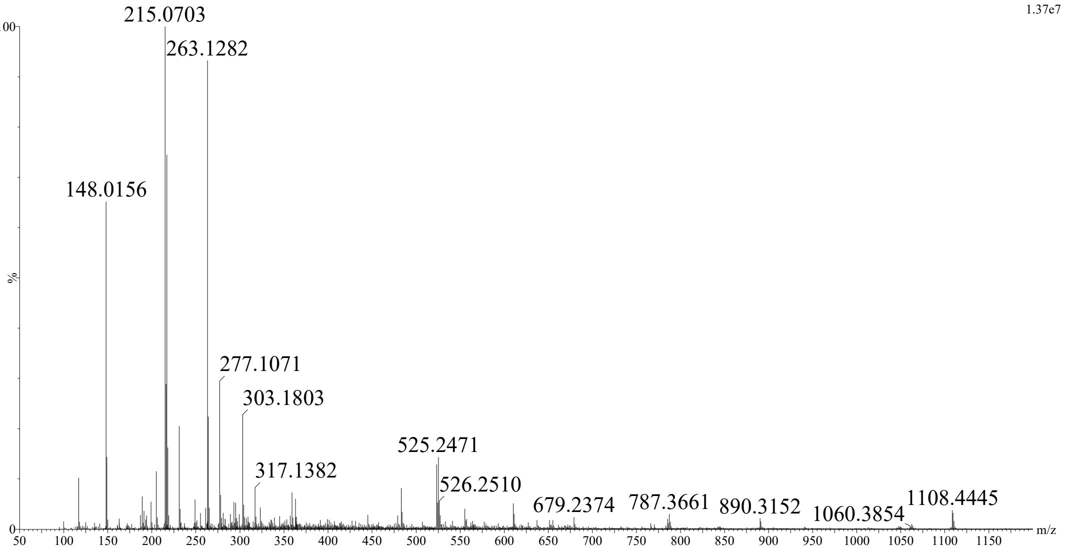

Figure S4. HRESIMS data of compound **1**

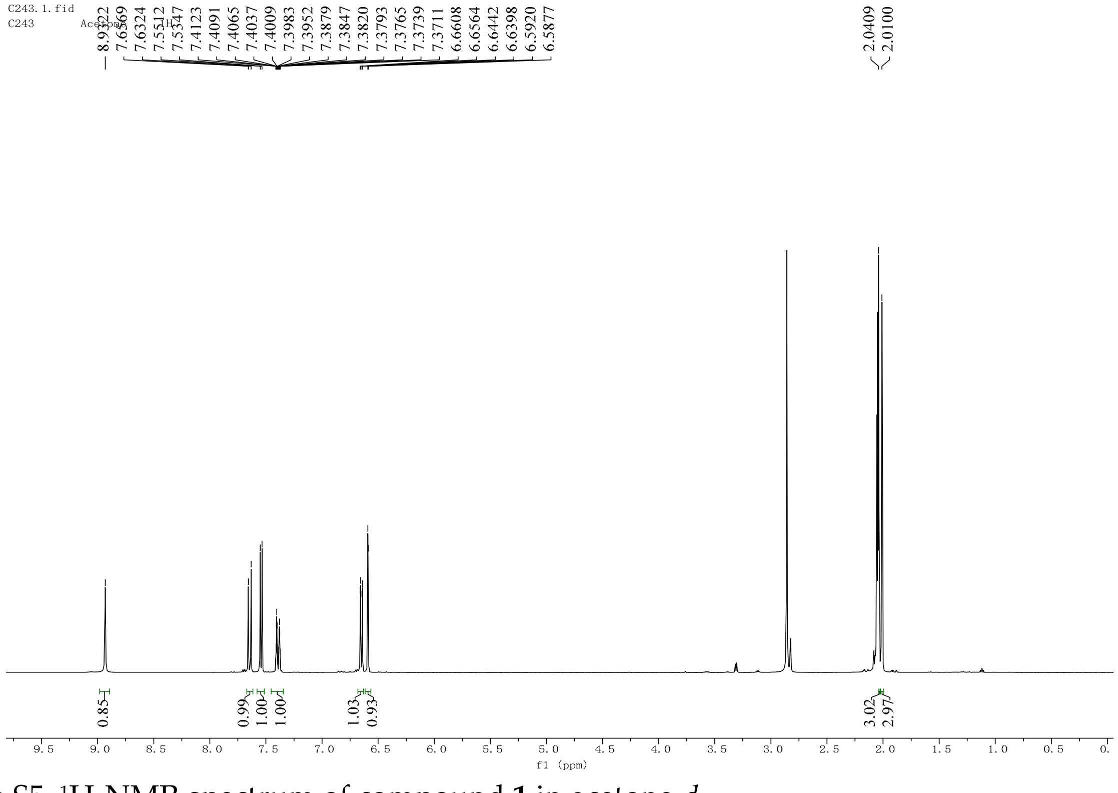

Figure S5. ^{1}H-NMR spectrum of compound **1** in acetone-d_6

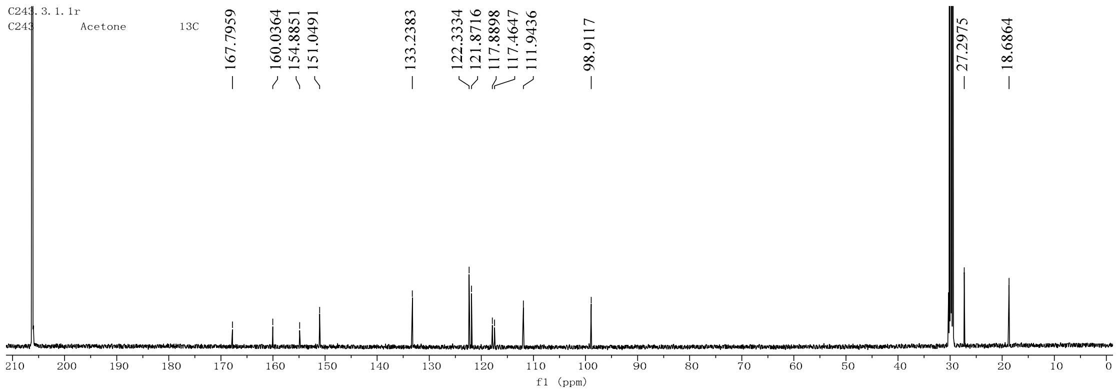

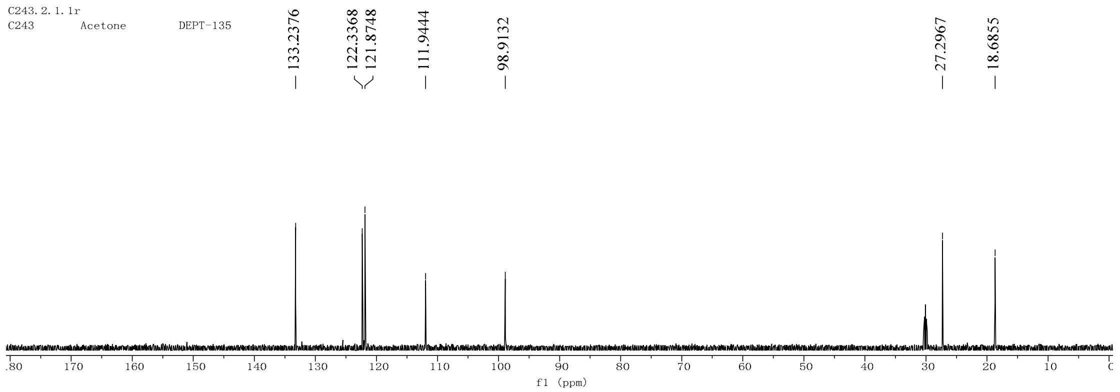

Figure S6. ^{13}C-NMR and DEPT-135 spectra of compound **1** in acetone-d_6

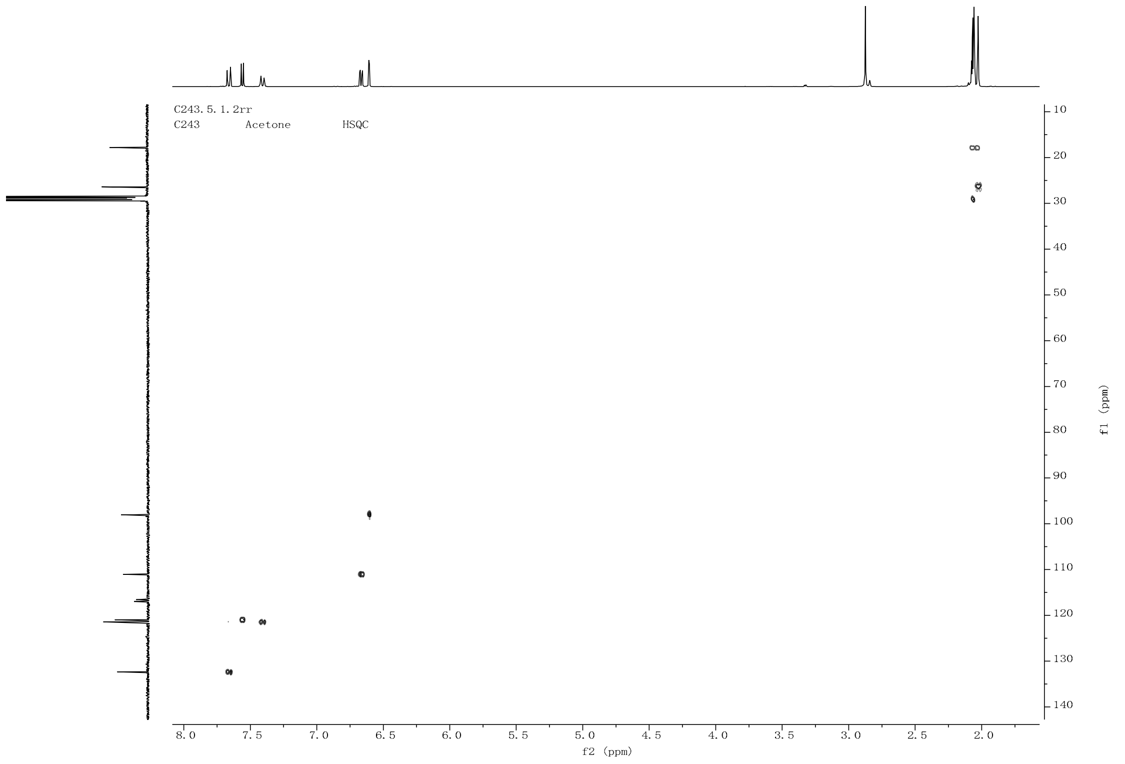

Figure S7. HSQC spectrum of compound **1**

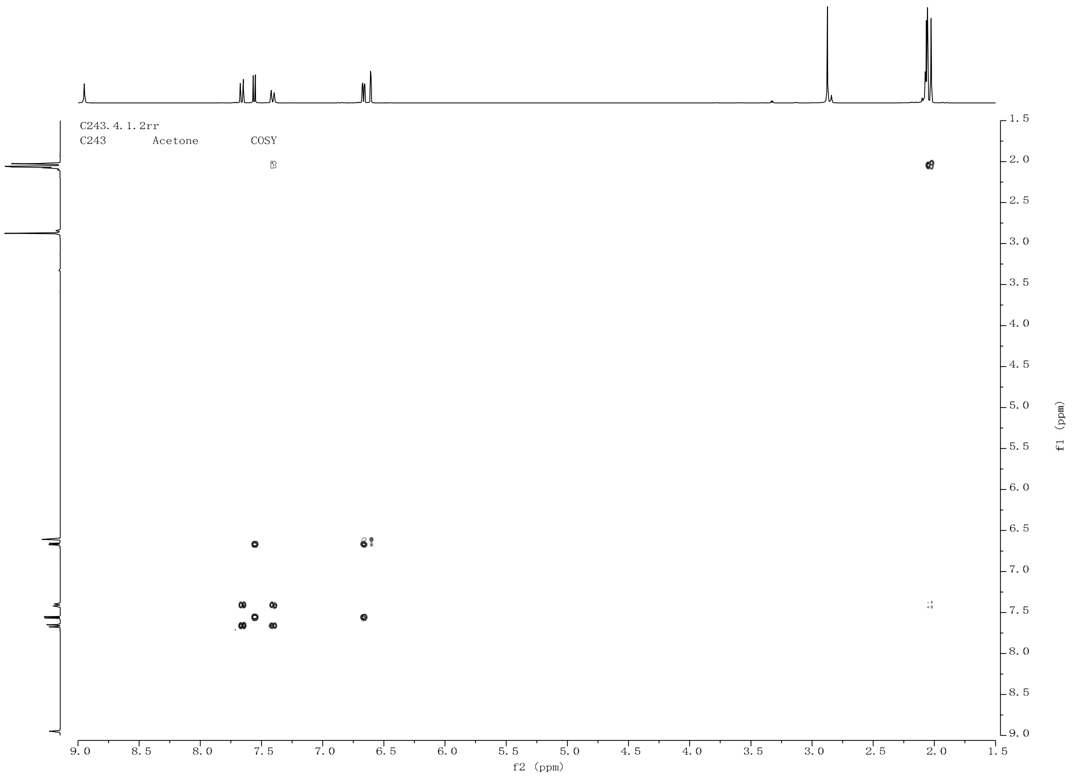

Figure S8. ^{1}H-^{1}H COSY spectrum of compound **1**

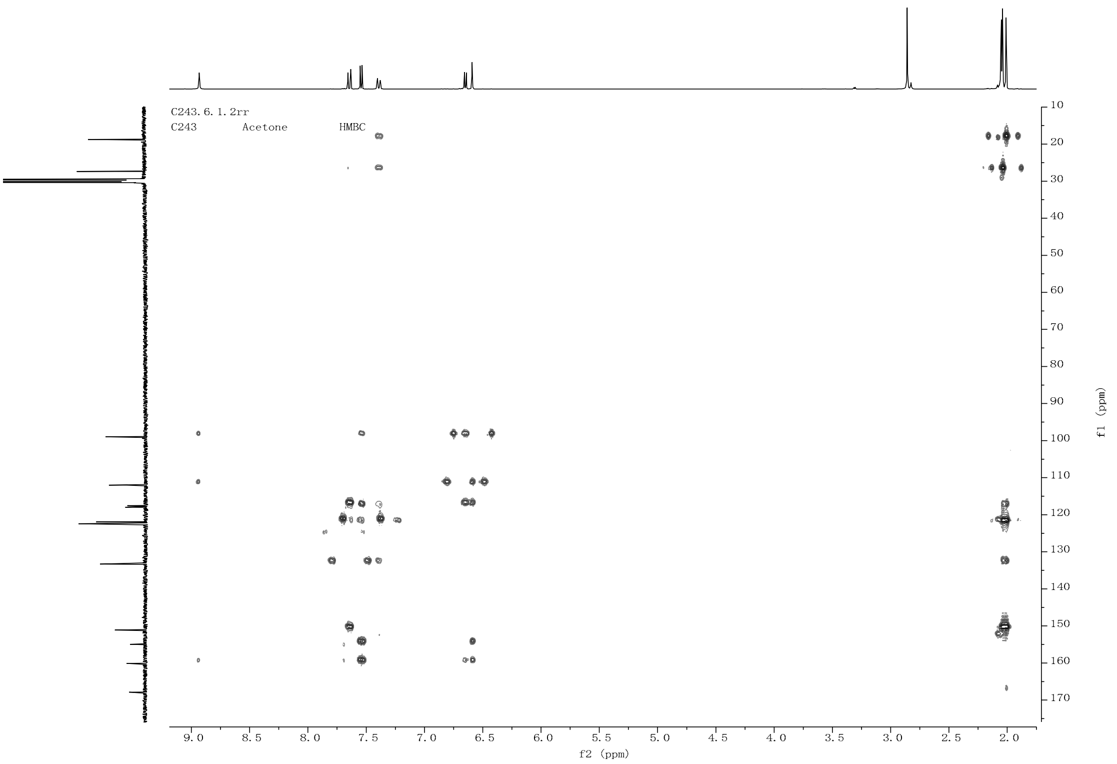

Figure S9. HMBC spectrum of compound **1**

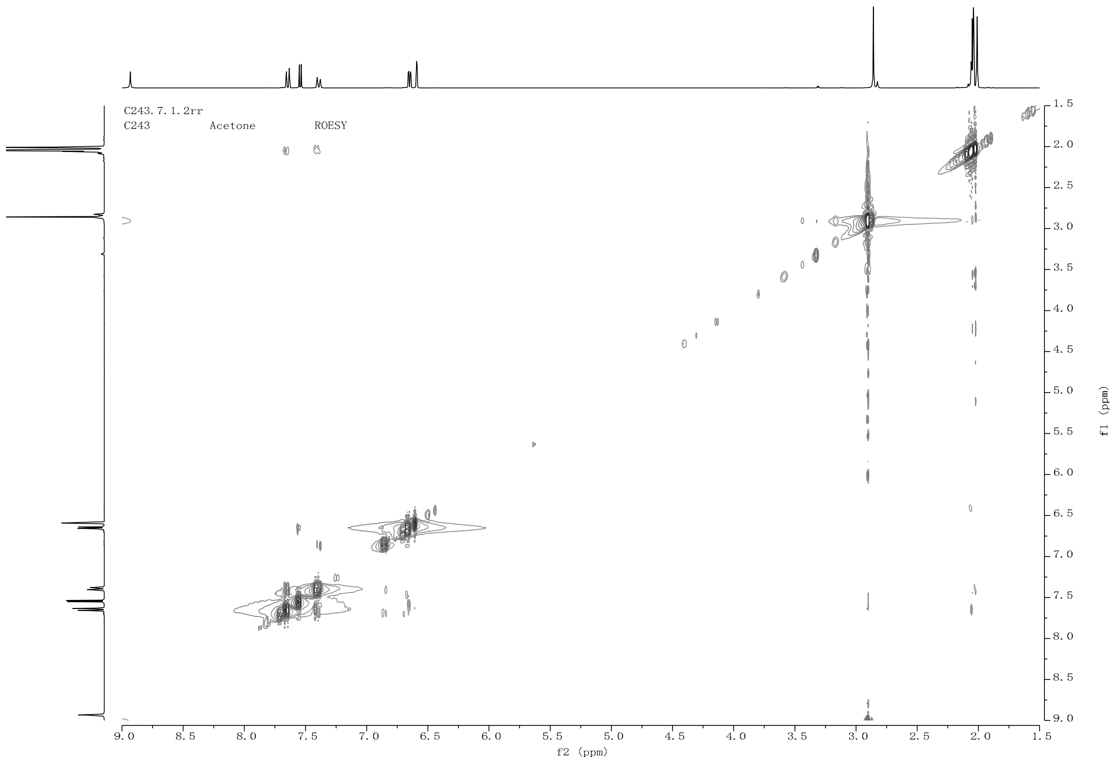

Figure S10. ROESY spectrum of compound **1**

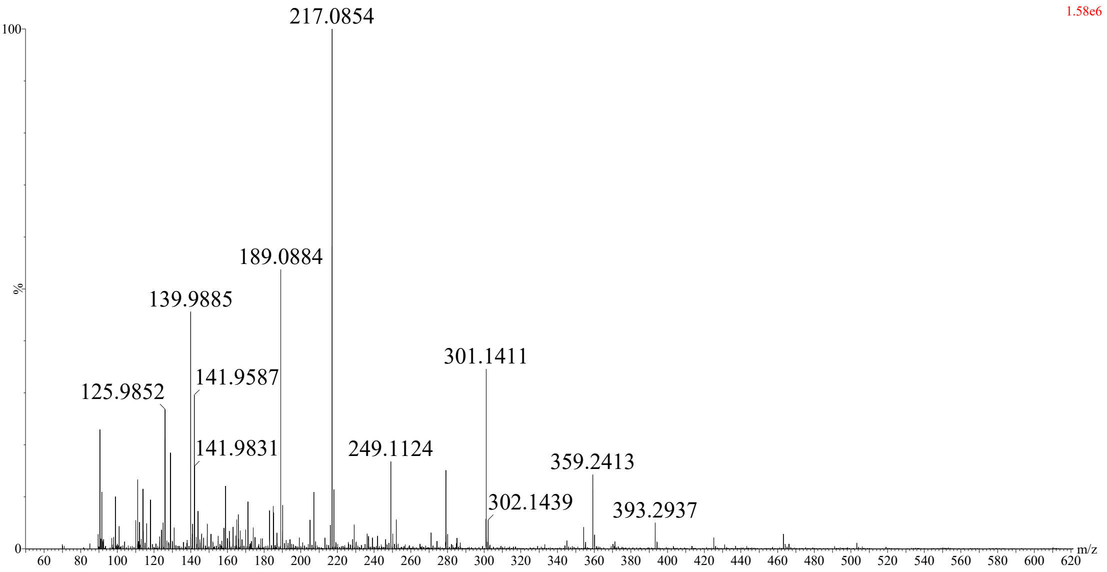

Figure S11. HRESIMS data of compound **2**

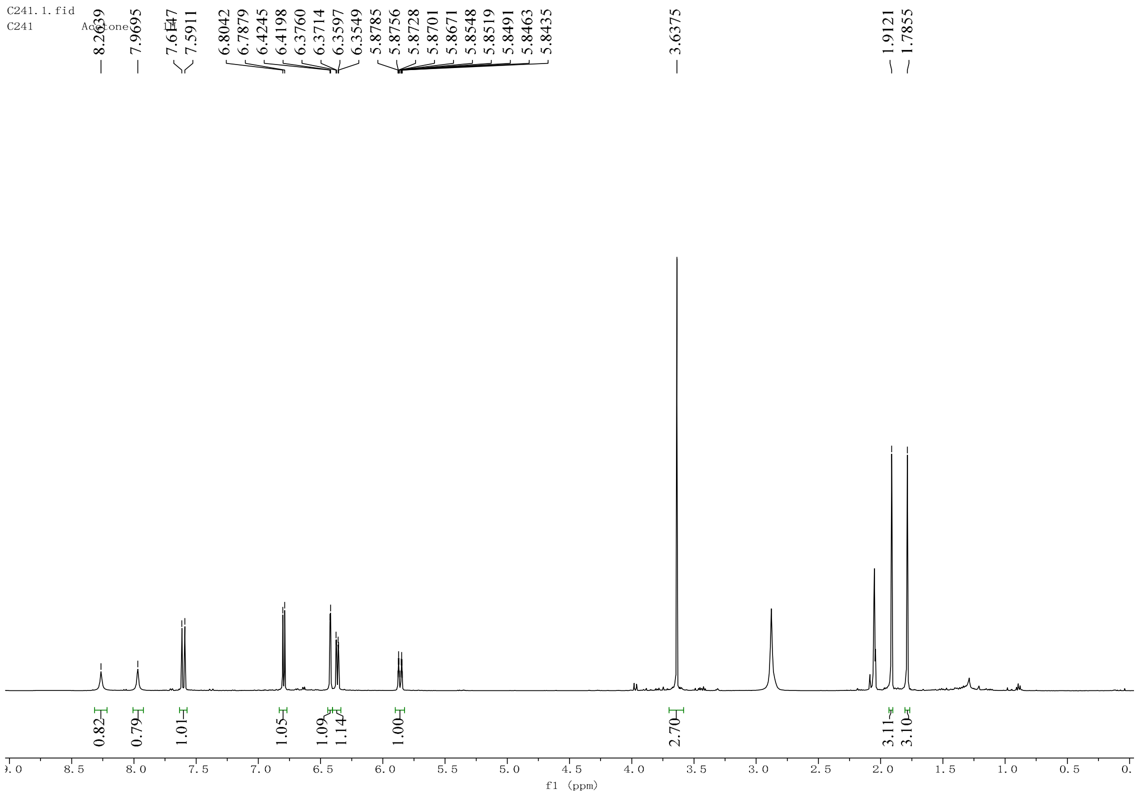

Figure S12. ^{1}H-NMR spectrum of compound **2** in acetone-d_6

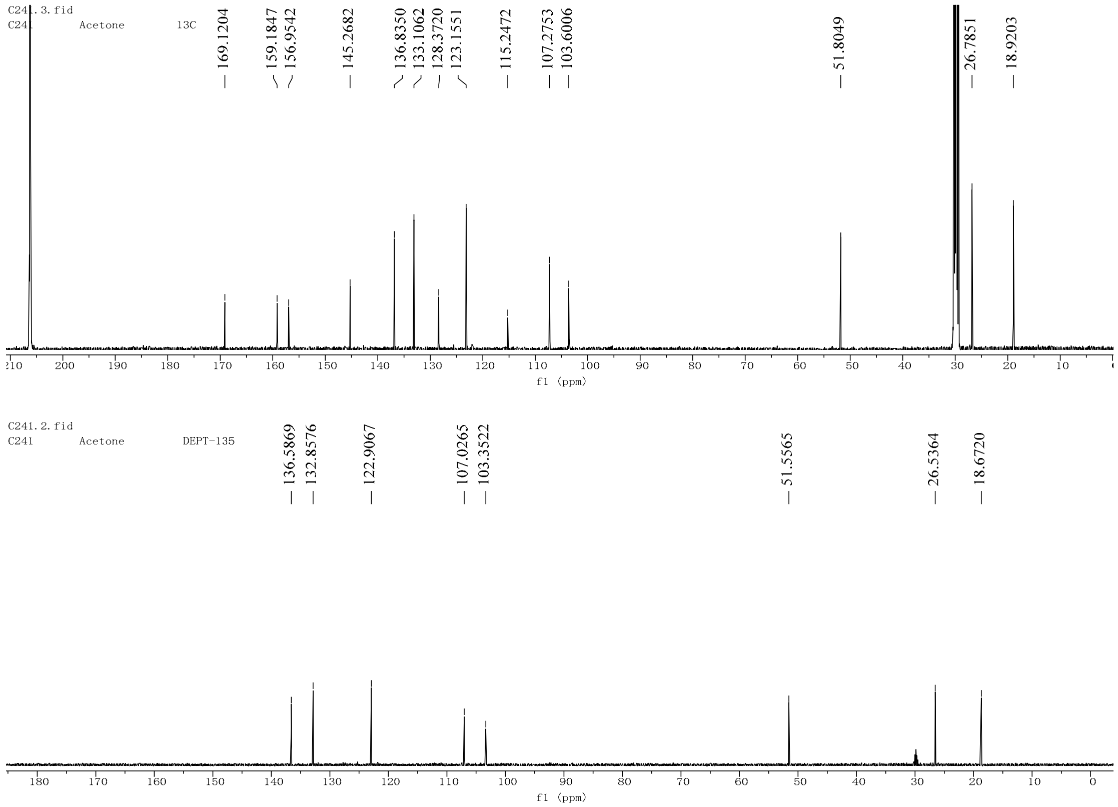

Figure S13. ^{13}C-NMR and DEPT-135 spectra of compound 2 in acetone-d_6

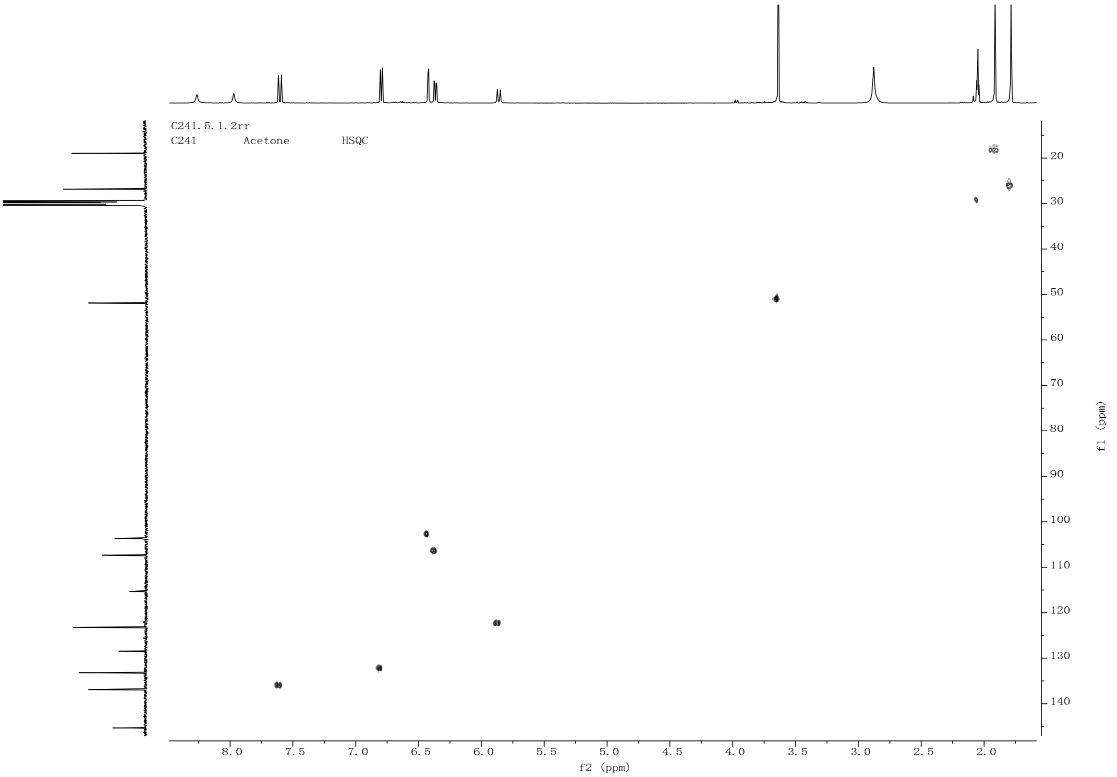

Figure S14. HSQC spectrum of compound **2**

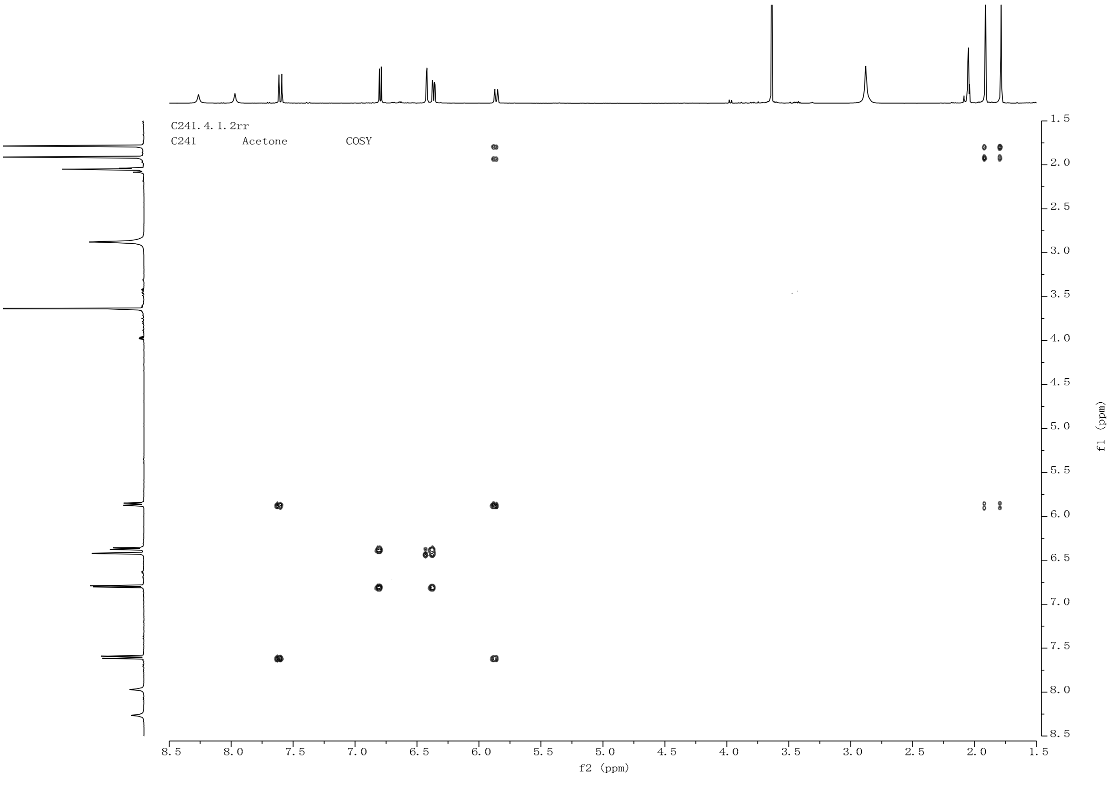

Figure S15. ^{1}H-^{1}H COSY spectrum of compound 2

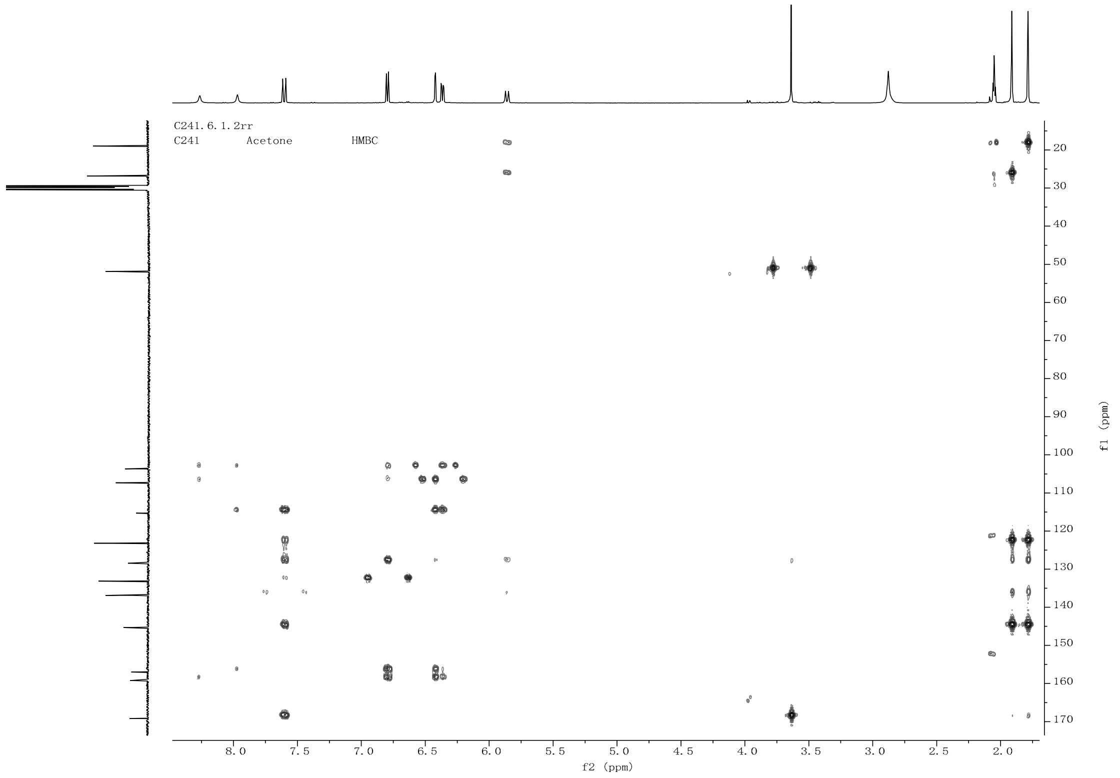

Figure S16. HMBC spectrum of compound **2**

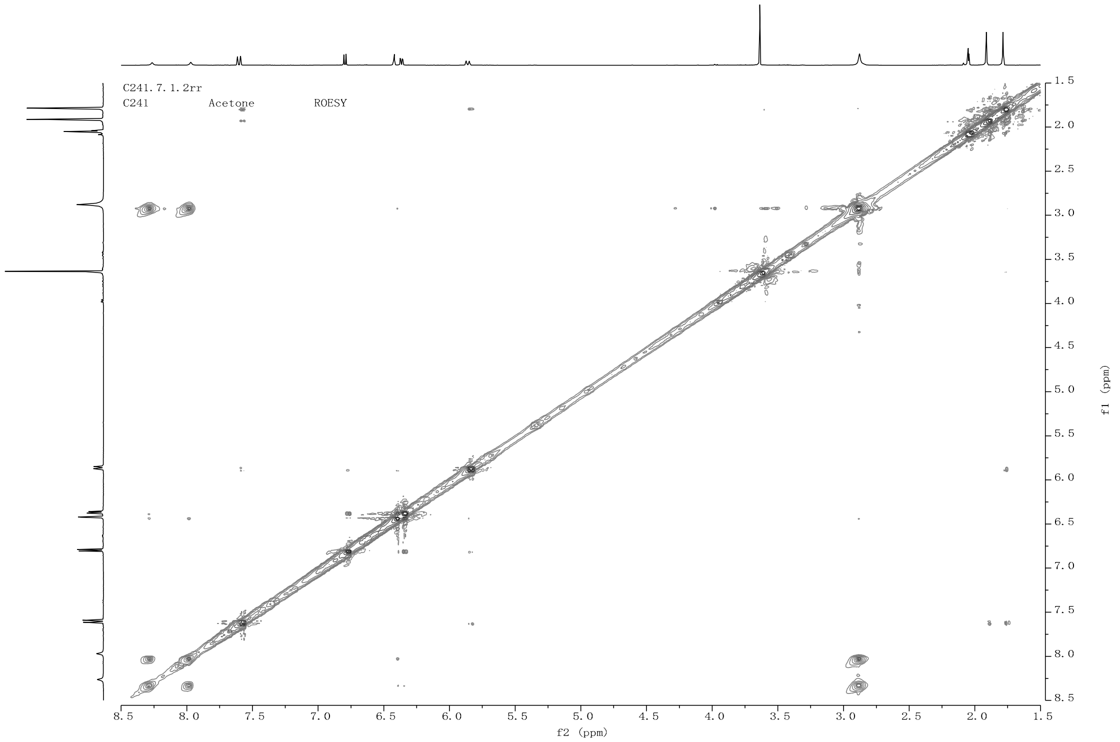

Figure S17. ROESY spectrum of compound **2**

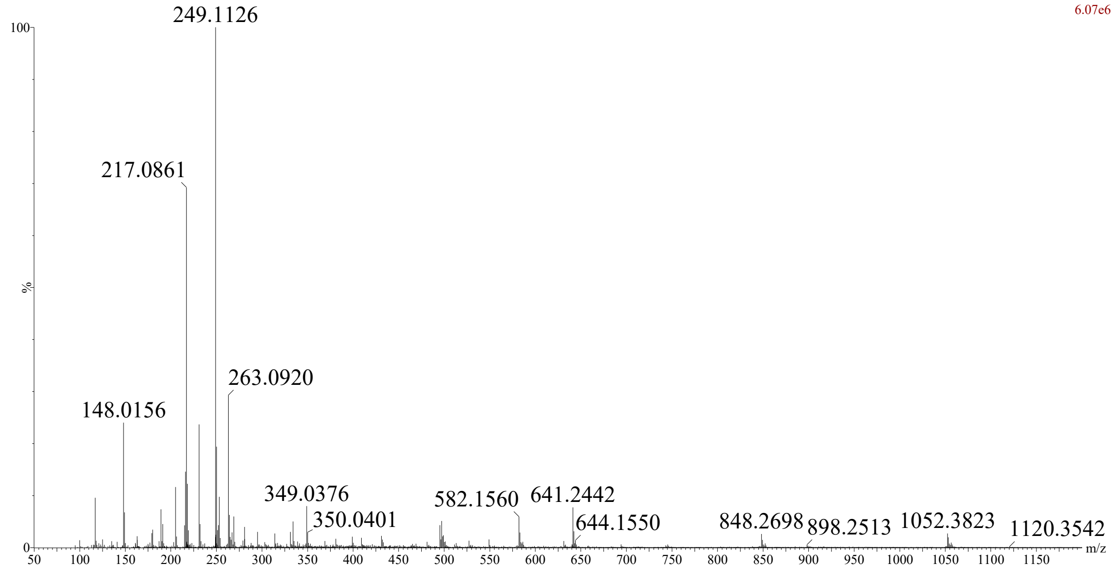

Figure S18. HRESIMS data of compound **3**

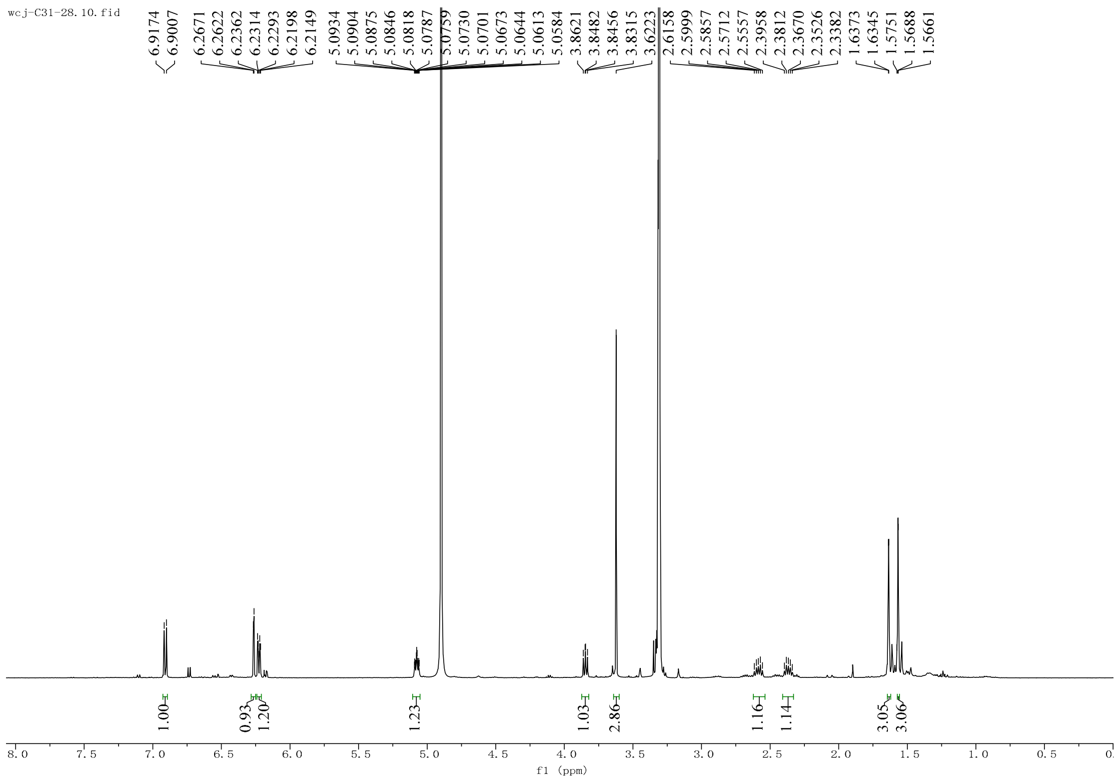

Figure S19. ^{1}H-NMR spectrum of compound **3** in CD_3OD

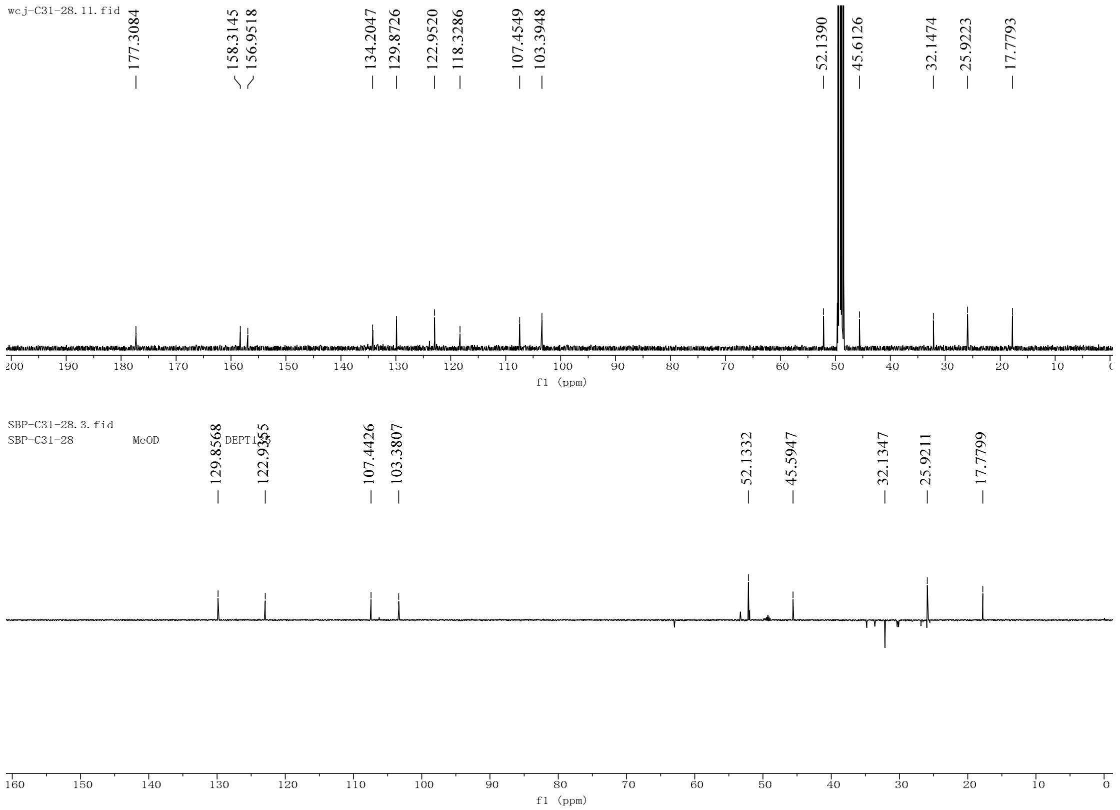

Figure S20. ^{13}C-NMR and DEPT-135 spectra of compound **3** in CD_3OD

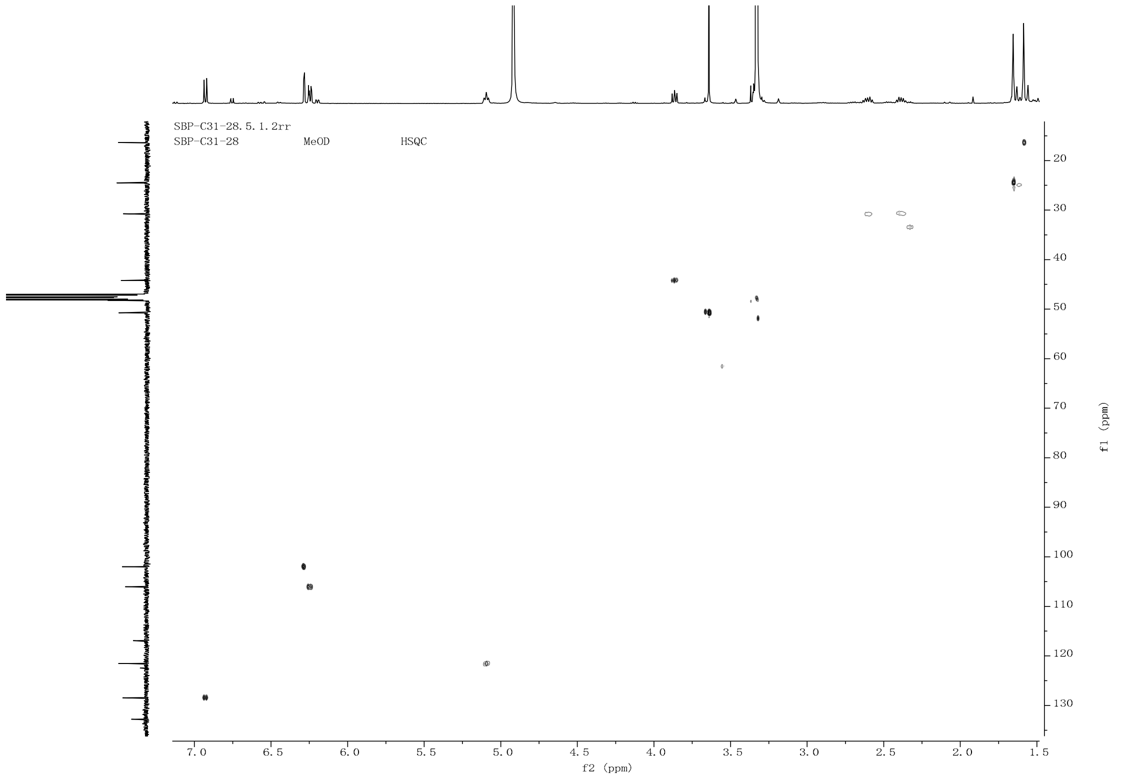

Figure S21. HSQC spectrum of compound **3**

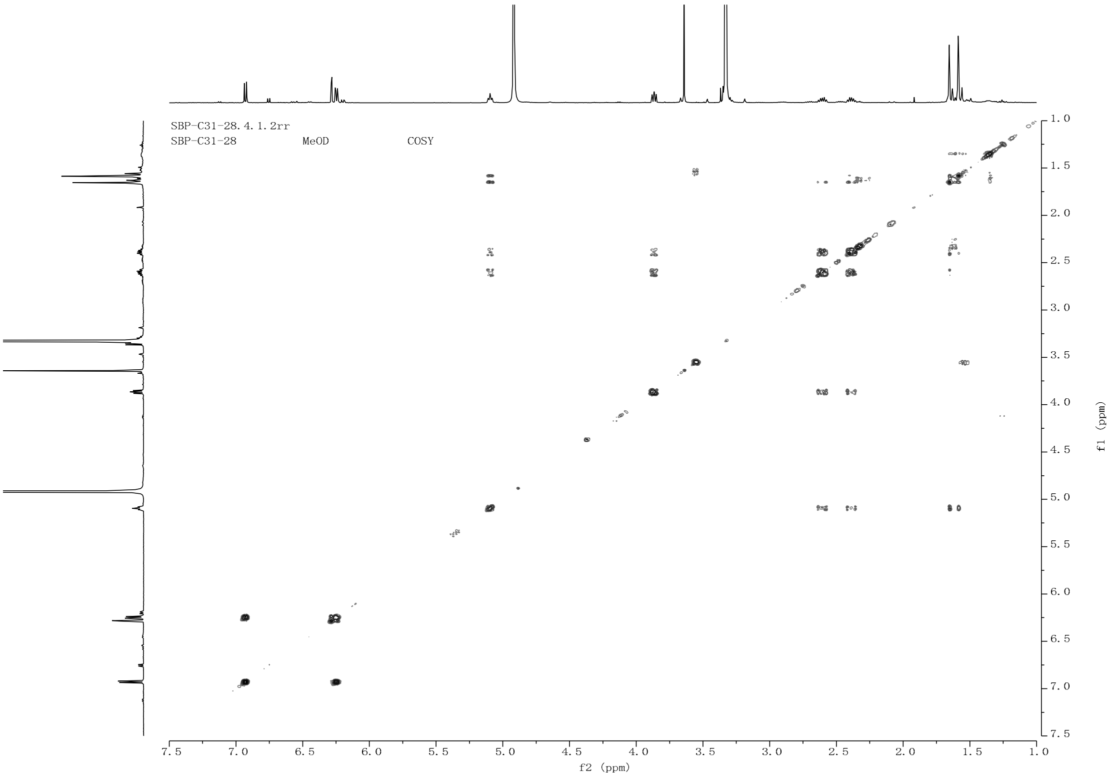

Figure S22. ^{1}H-^{1}H COSY spectrum of compound **3**

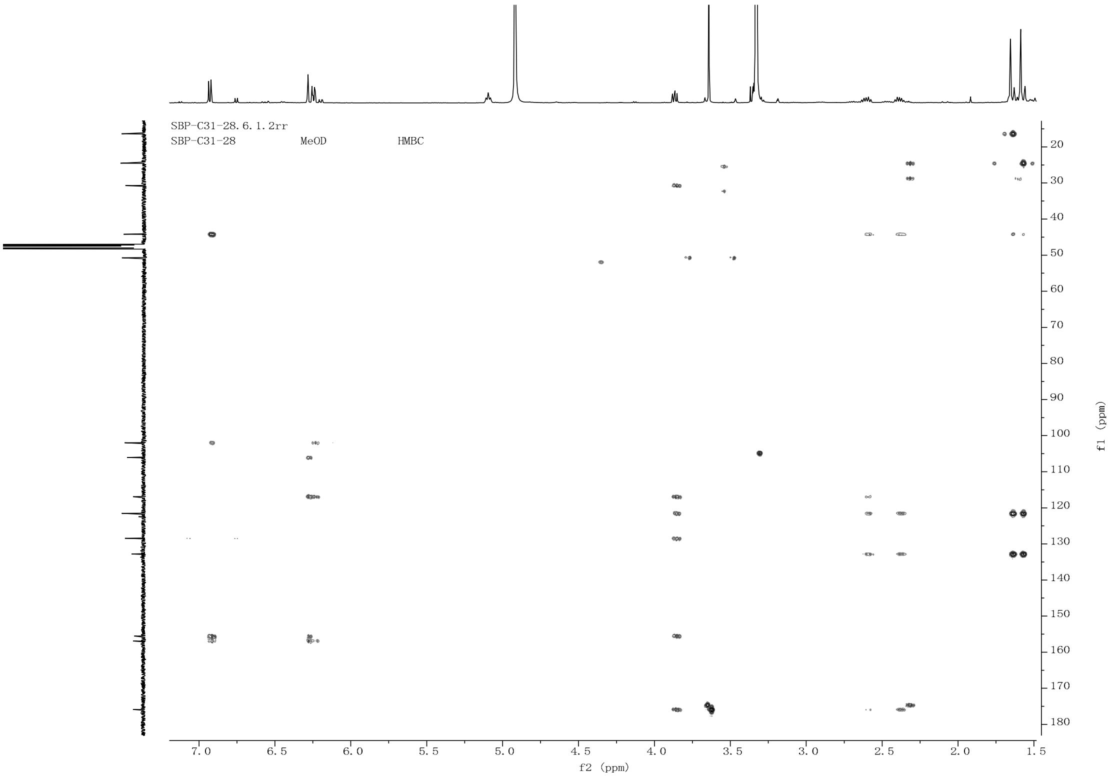

Figure S23. HMBC spectrum of compound **3**

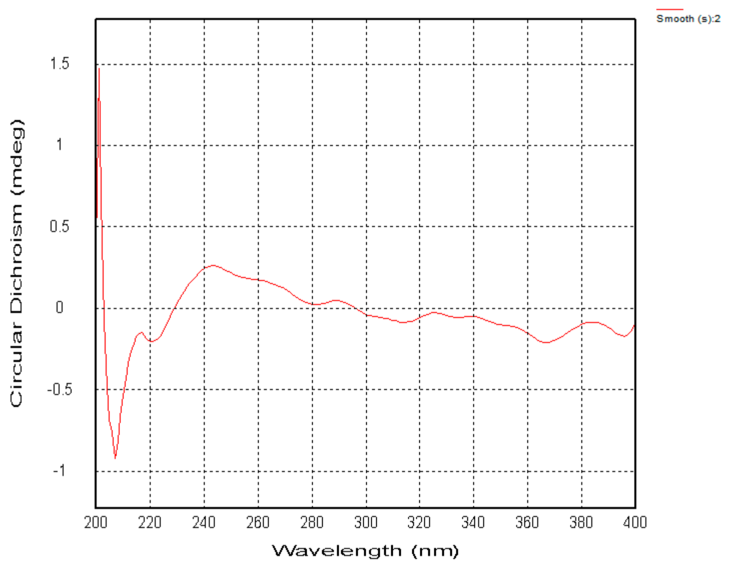

Figure S24. CD spectrum of compound **3**

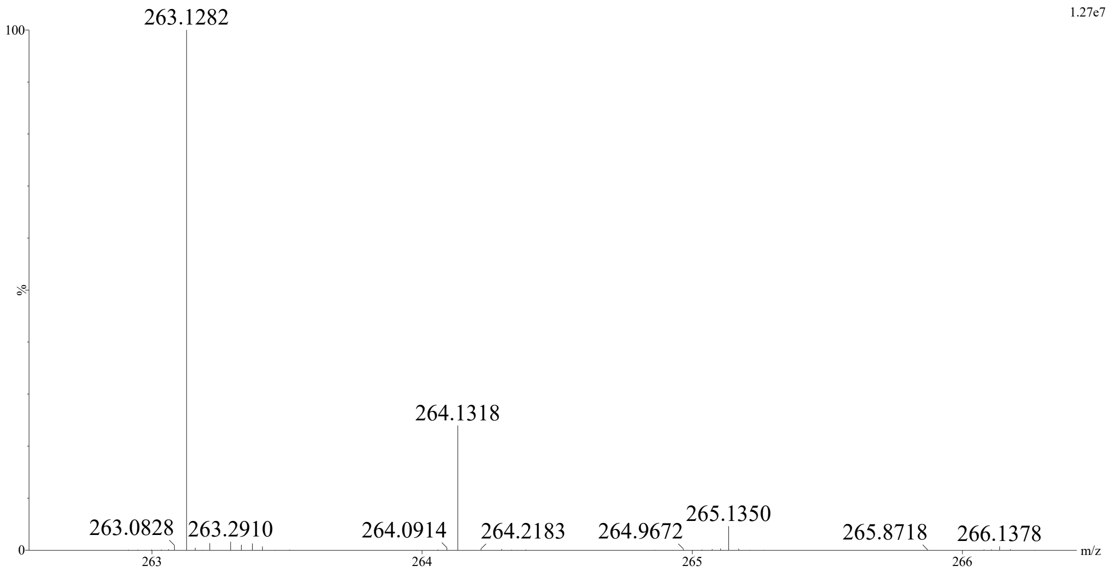

Figure S25. HRESIMS data of compound **4**

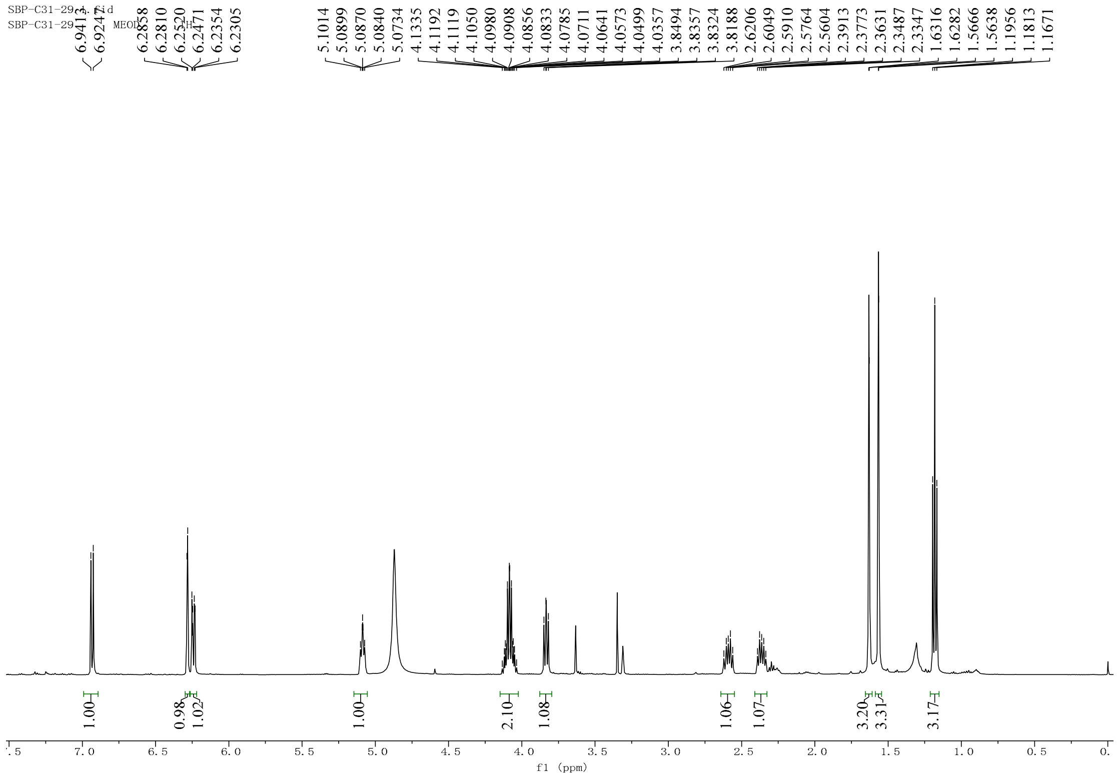

Figure S26. ^{1}H-NMR spectrum of compound **4** in CD_3OD

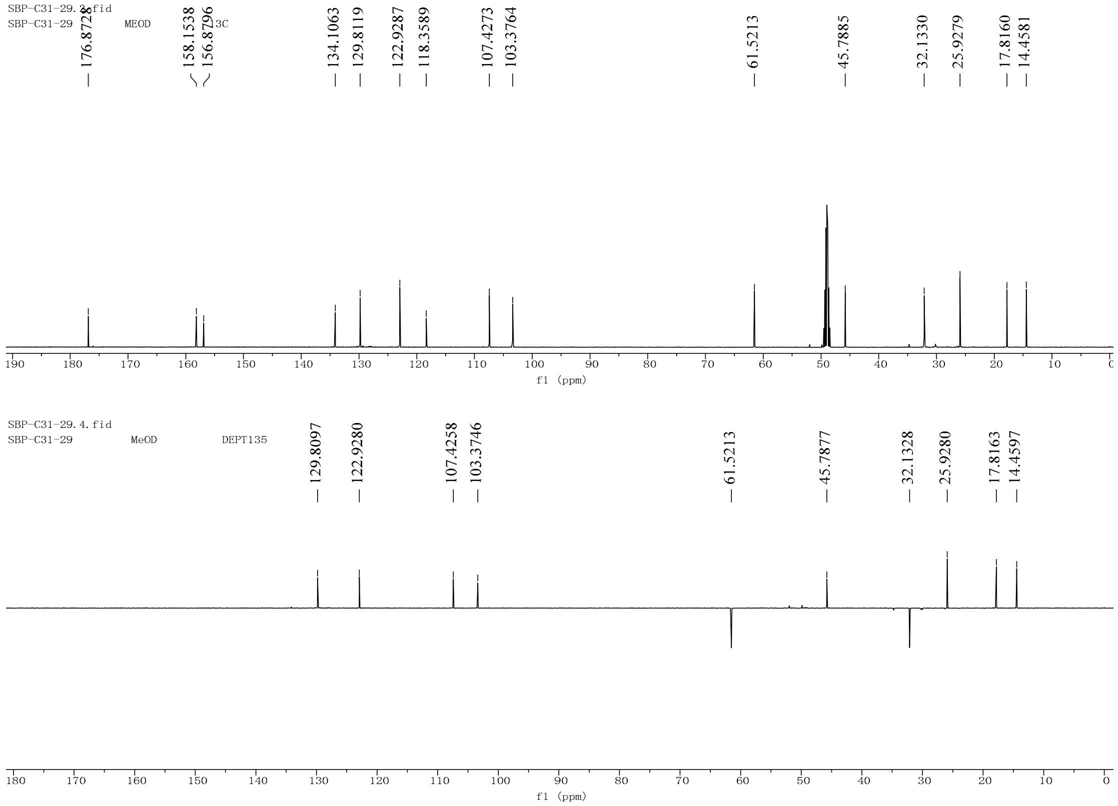

Figure S27. ^{13}C-NMR and DEPT-135 spectra of compound **4** in CD_3OD

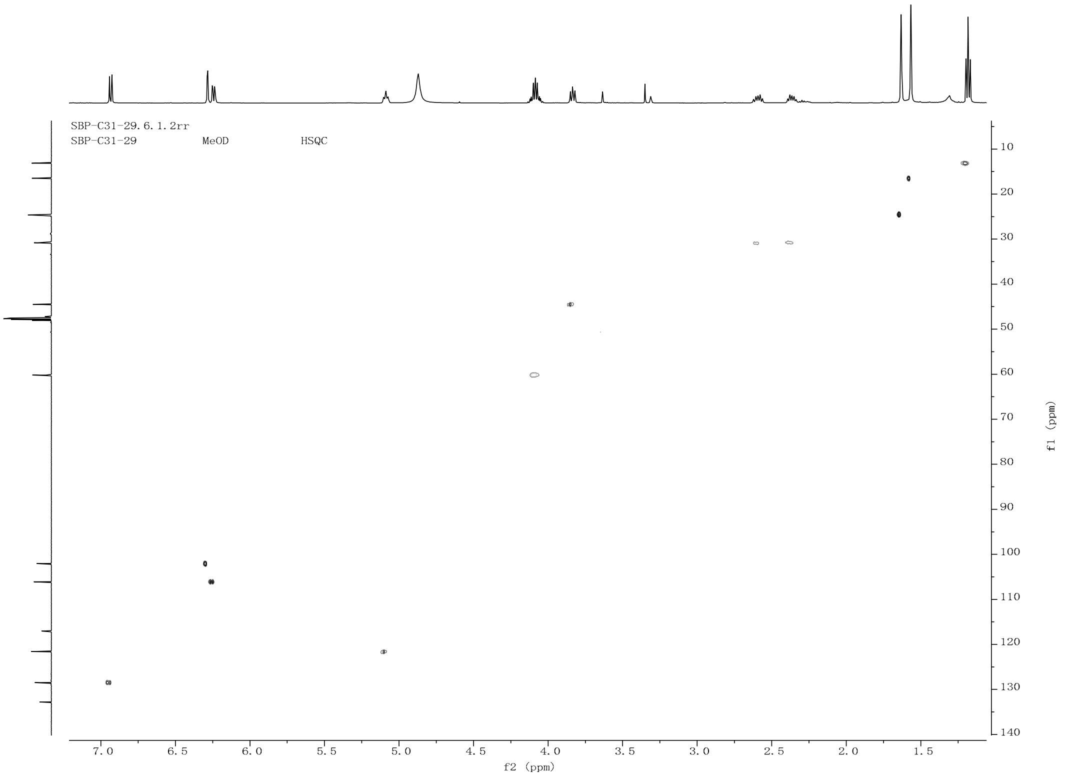

Figure S28. HSQC spectrum of compound **4**

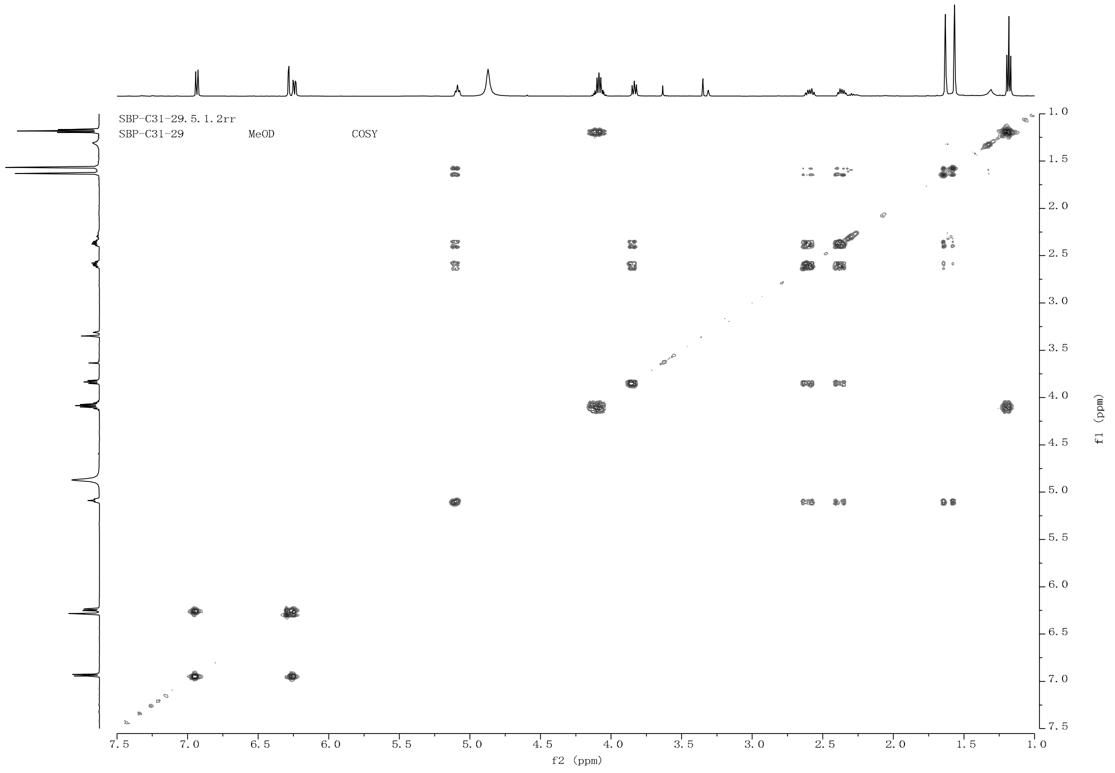

Figure S29. ^{1}H-^{1}H COSY spectrum of compound **4**

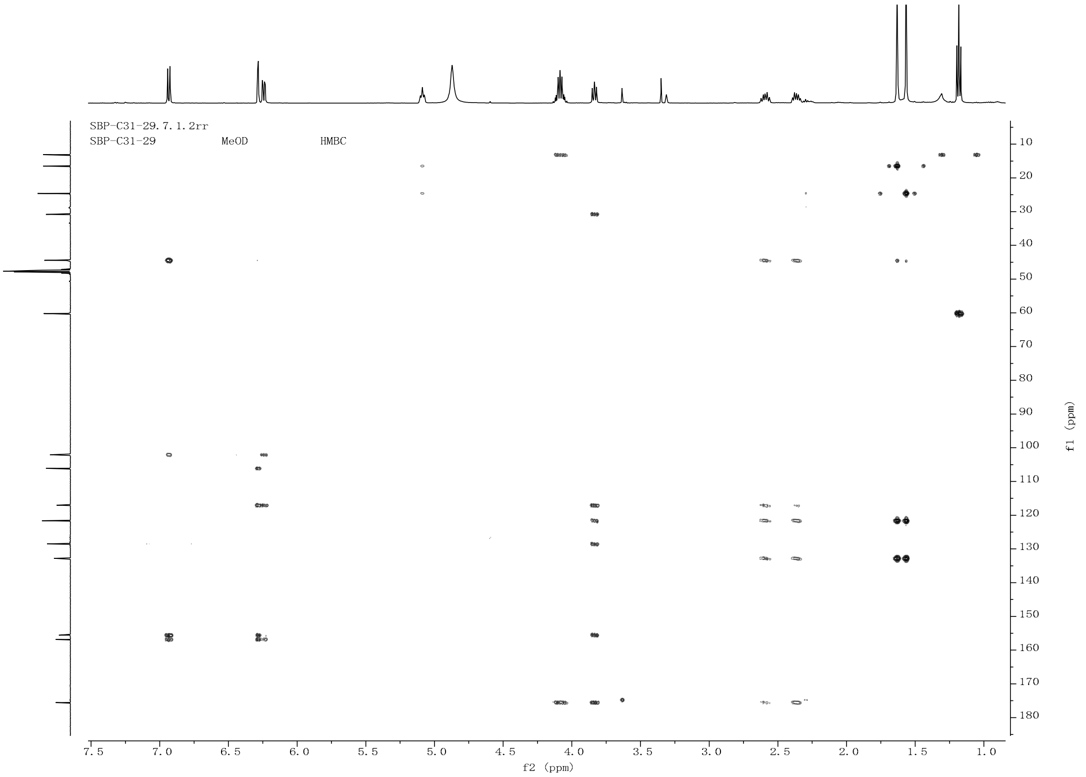

Figure S30. HMBC spectrum of compound **4**

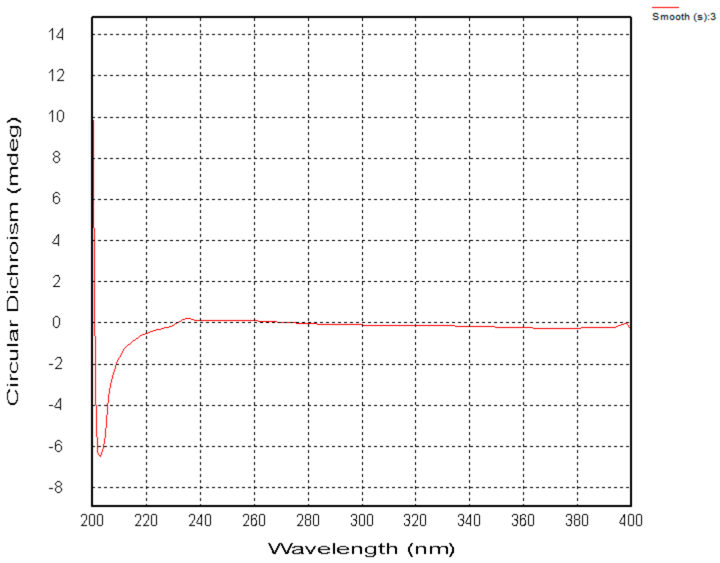

Figure S31. CD spectrum of compound **4**

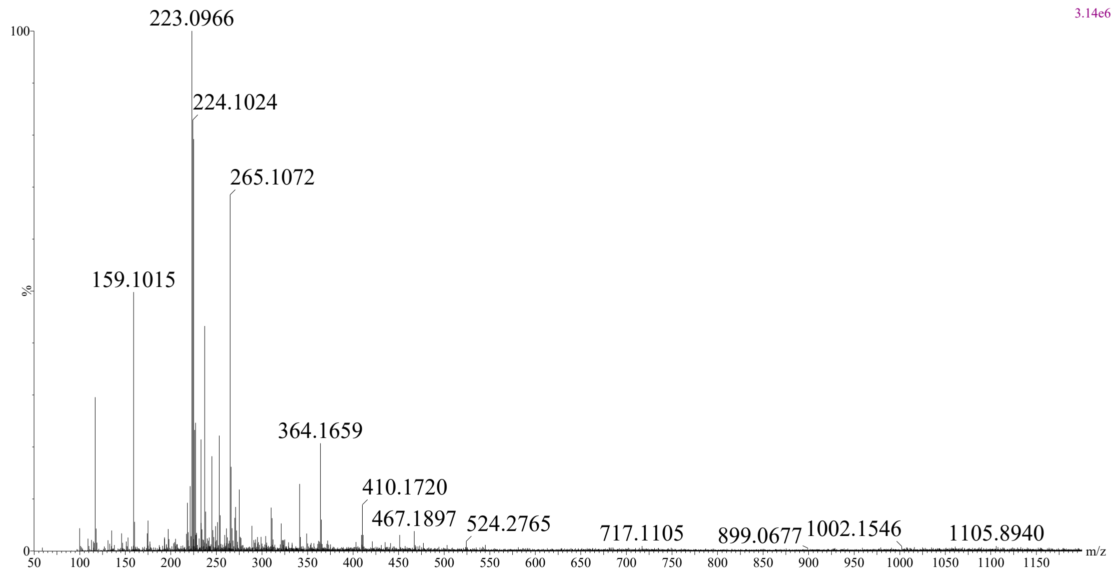

Figure S32. HRESIMS data of compound **5**

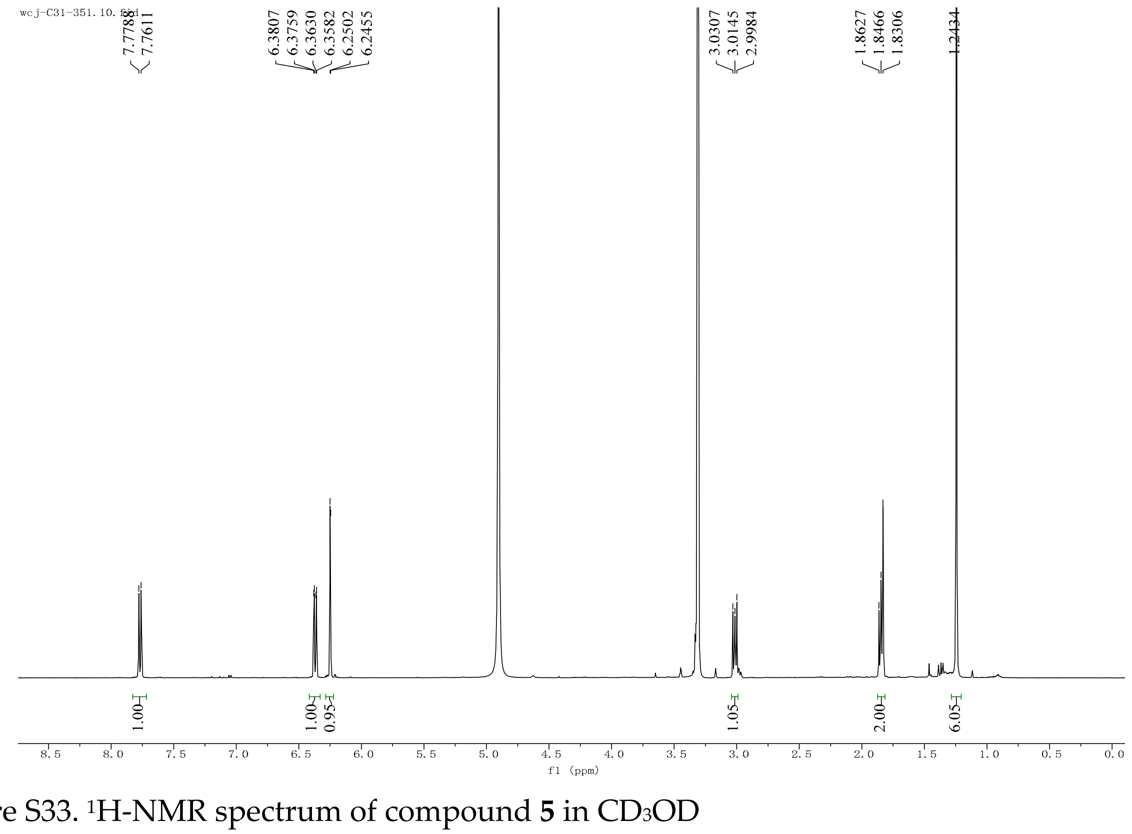

Figure S33. ^{1}H-NMR spectrum of compound **5** in CD_3OD

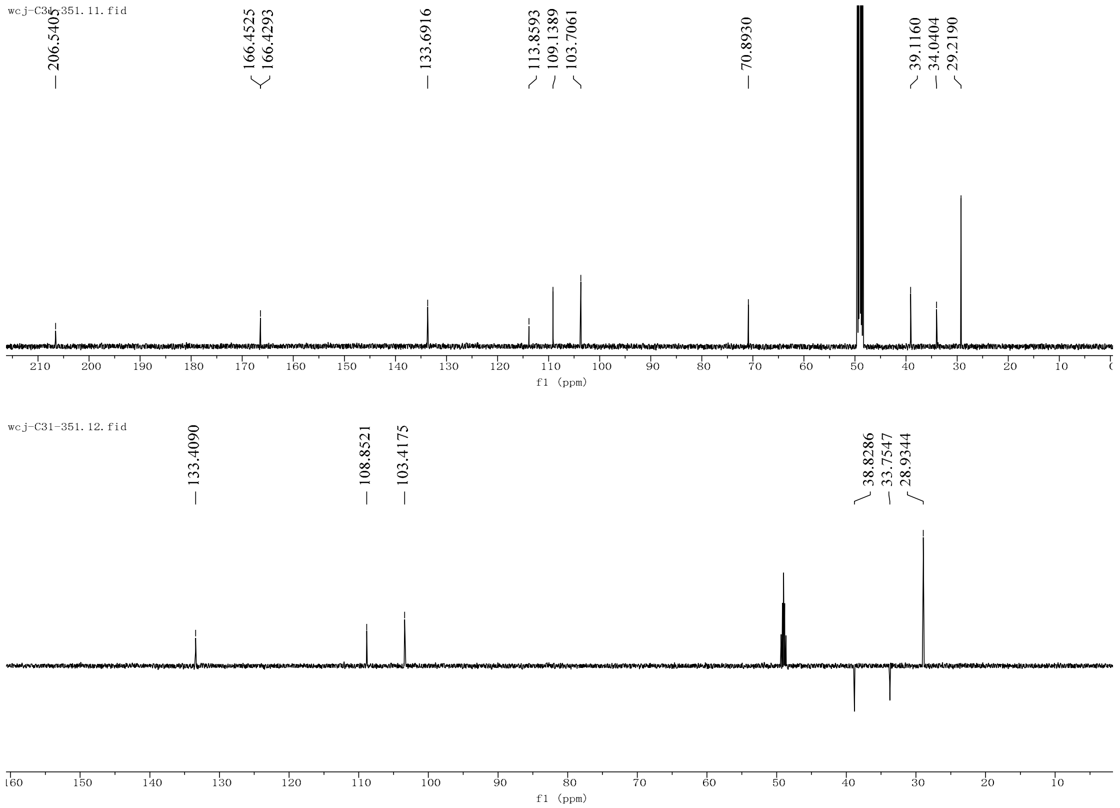

Figure S34. ^{13}C-NMR and DEPT-135 spectra of compound **5** in CD_3OD

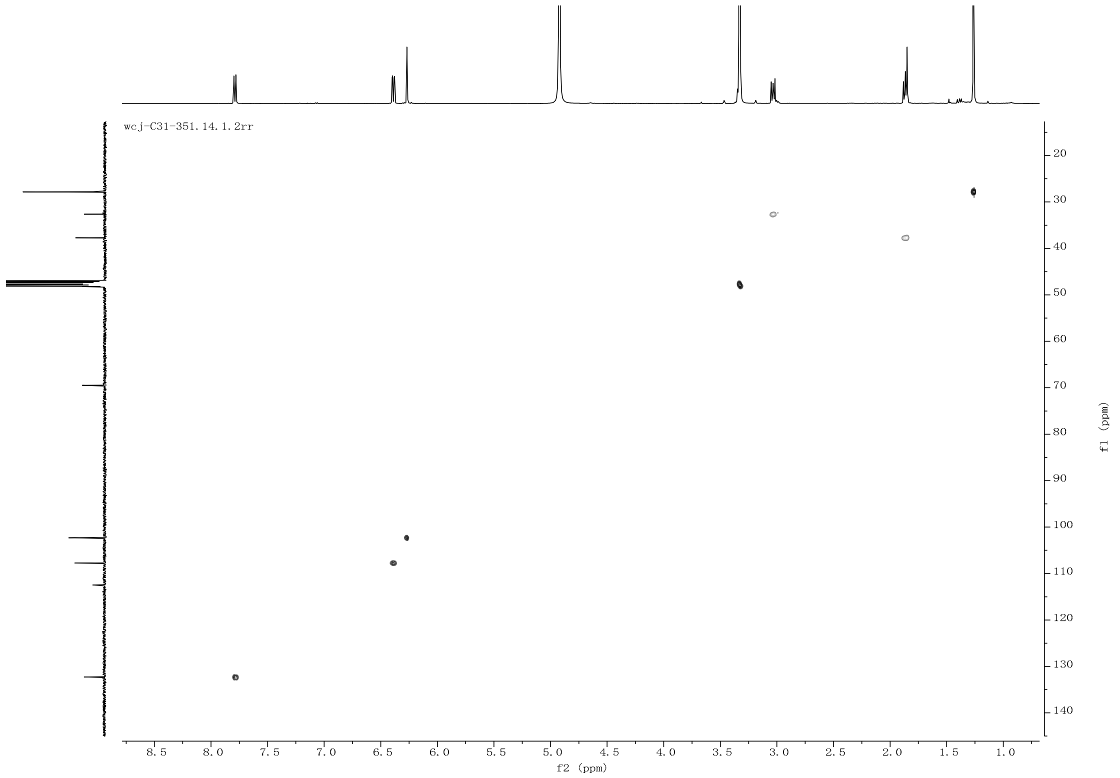

Figure S35. HSQC spectrum of compound **5**

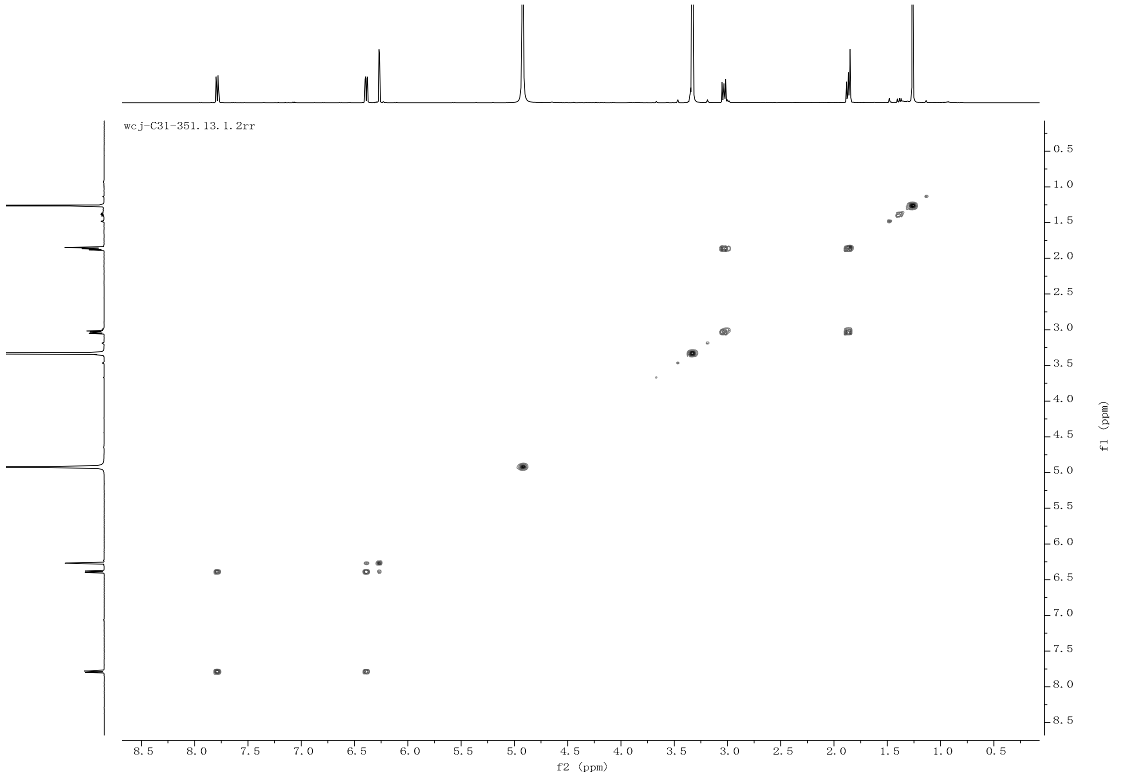

Figure S36. ^{1}H-^{1}H COSY spectrum of compound **5**

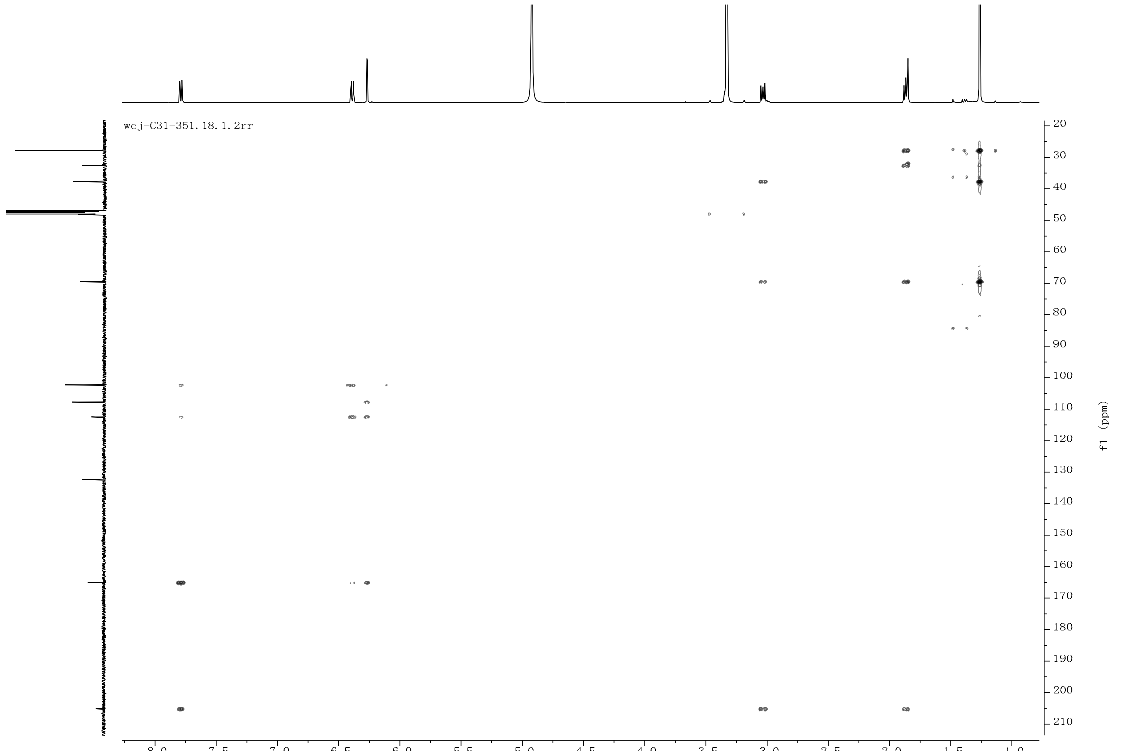

Figure S37. HMBC spectrum of compound **5**

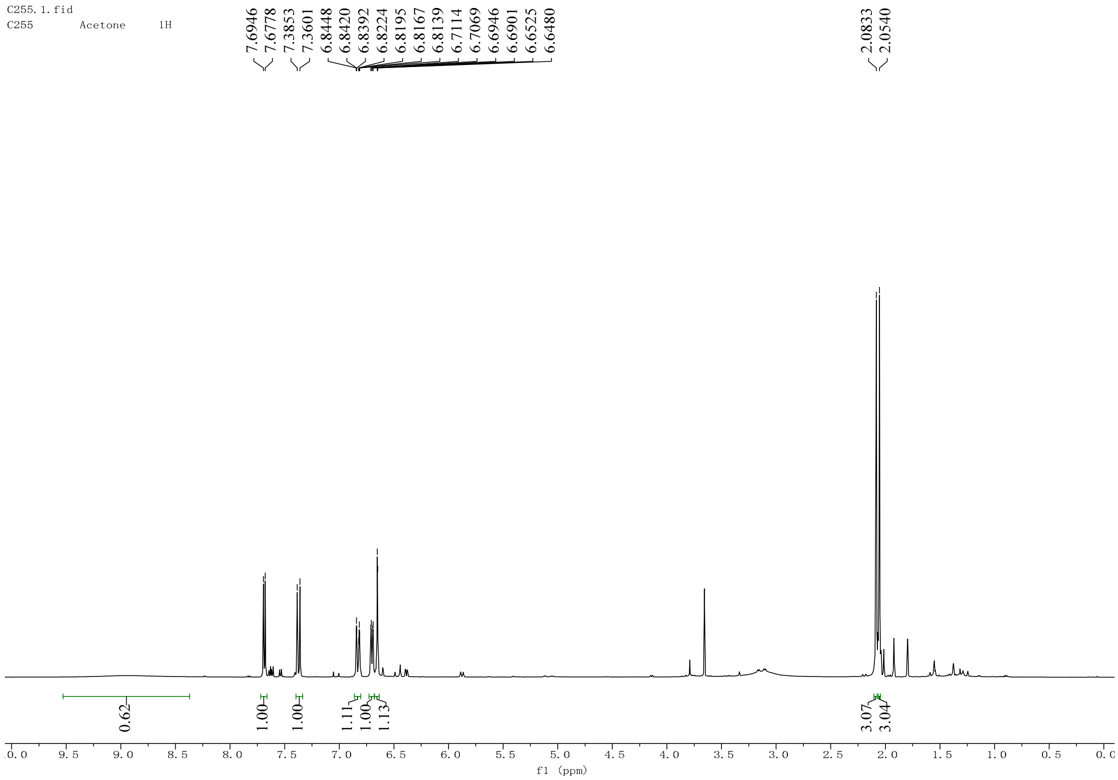

Figure S38. ^{1}H-NMR spectrum of compound **6** in acectone-d_6

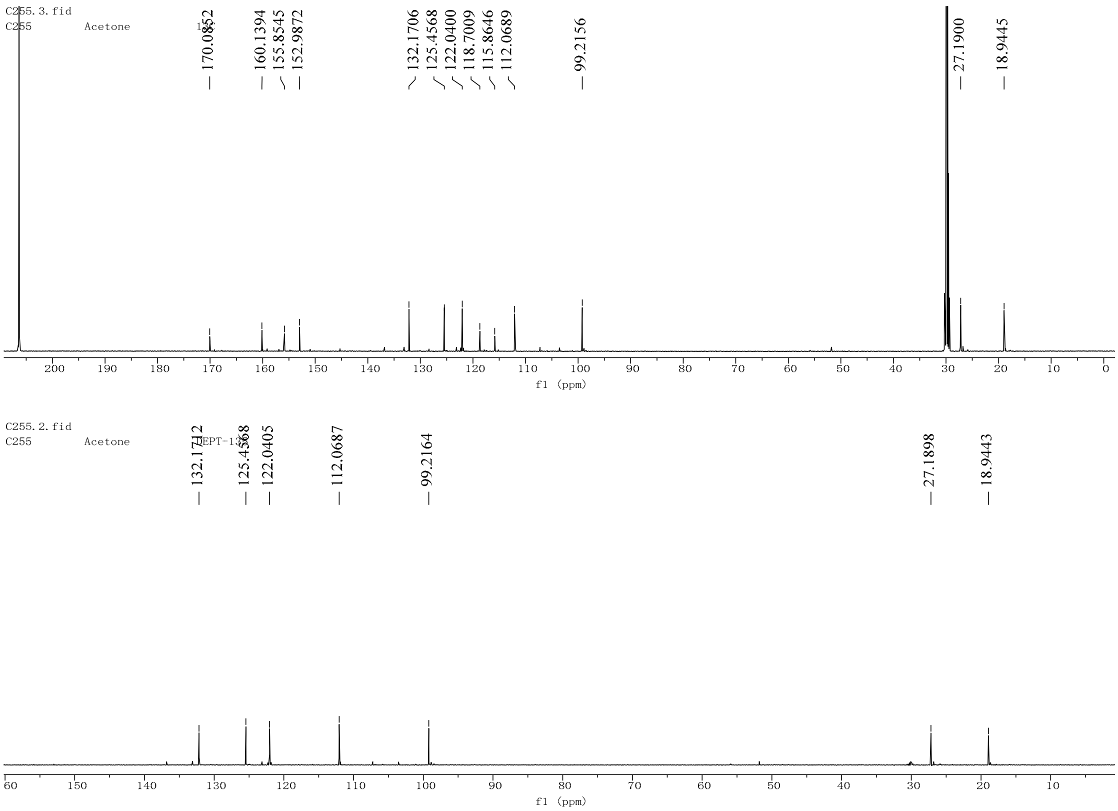

Figure S39. ^{13}C-NMR and DEPT-135 spectra of compound **6** in acectone-d_6

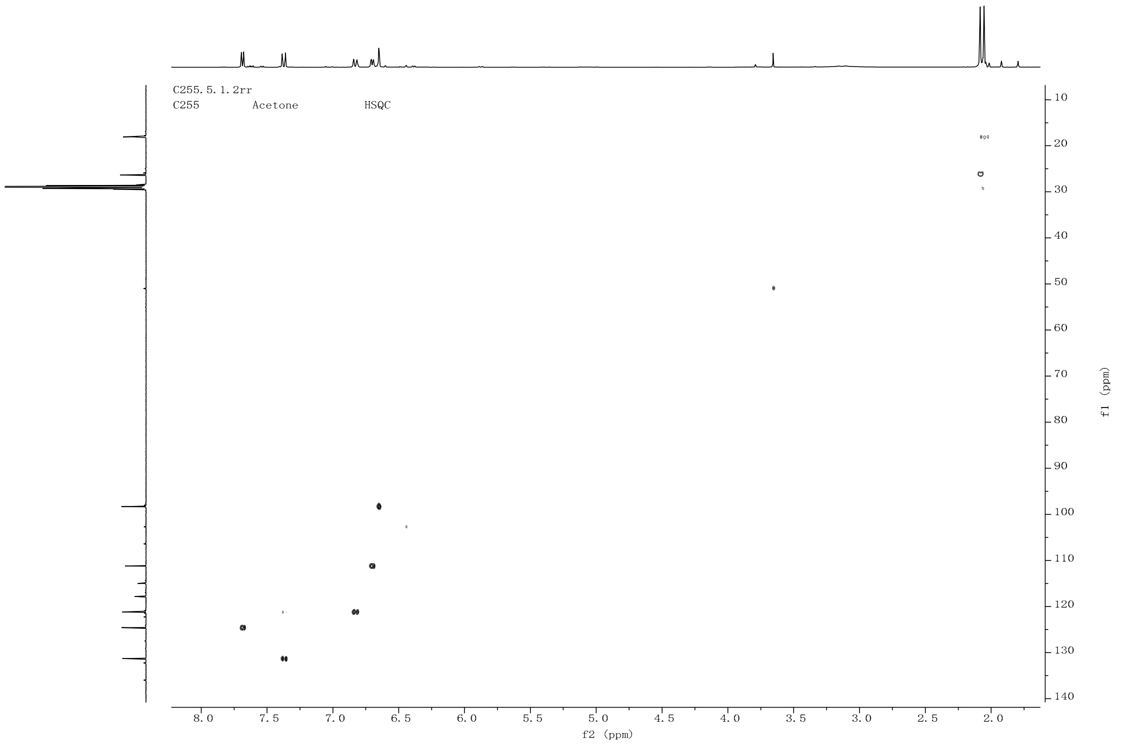

Figure S40. HSQC spectrum of compound **6**

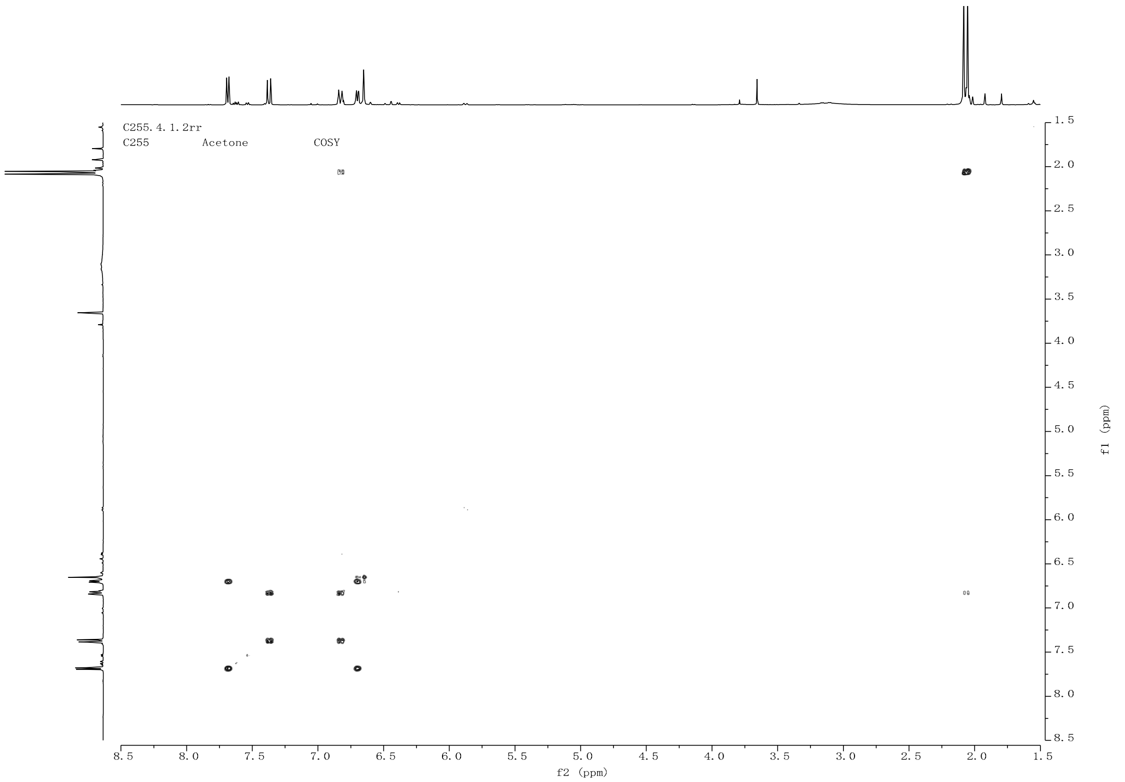

Figure S41. ^{1}H-^{1}H COSY spectrum of compound **6**

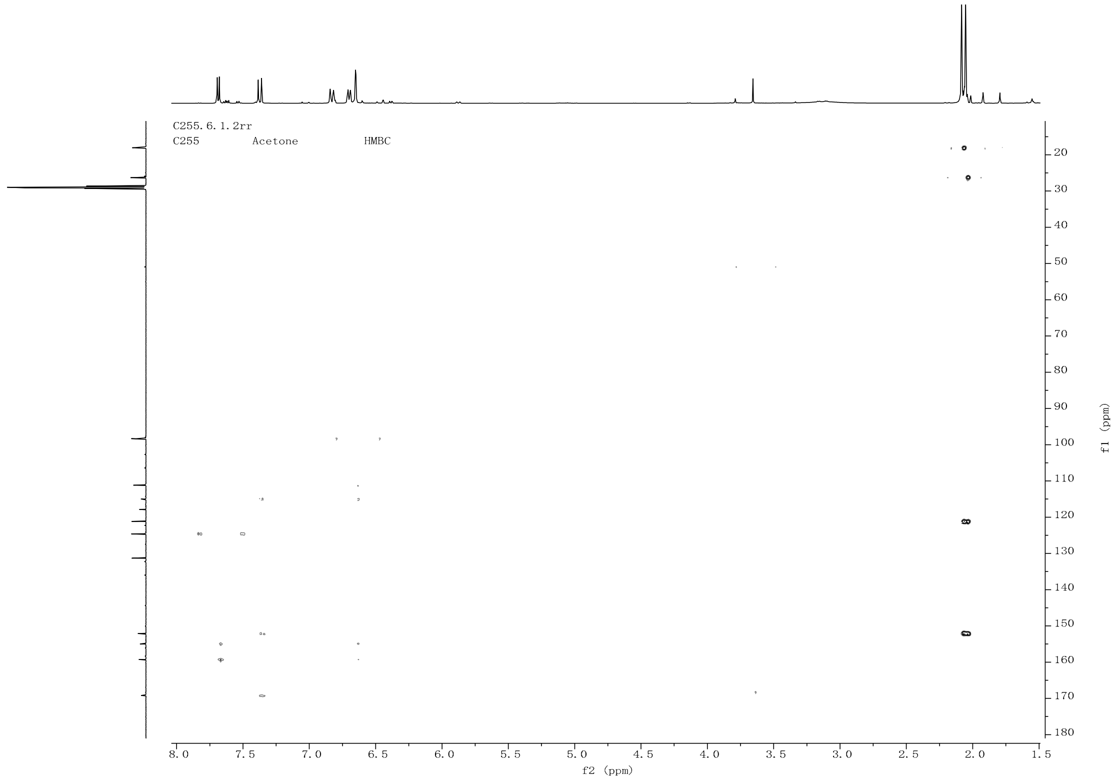

Figure S42. HMBC spectrum of compound **6**

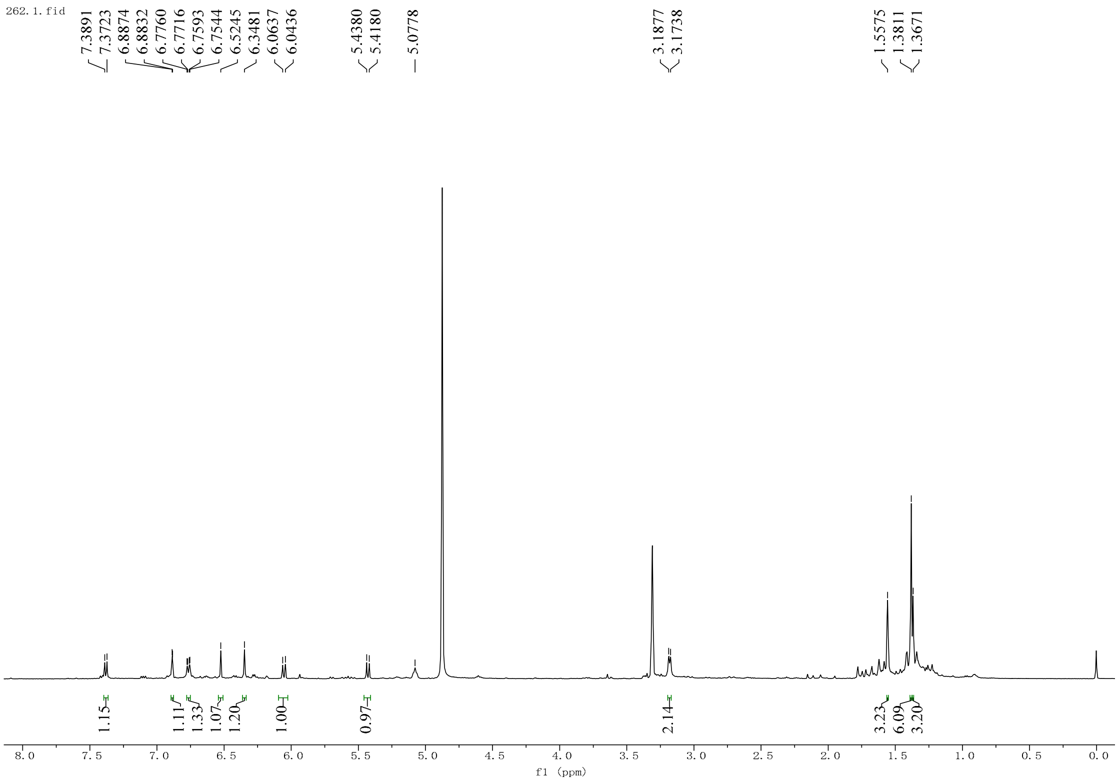

Figure S43. ^{1}H-NMR spectrum of compound **14** in CD_3OD

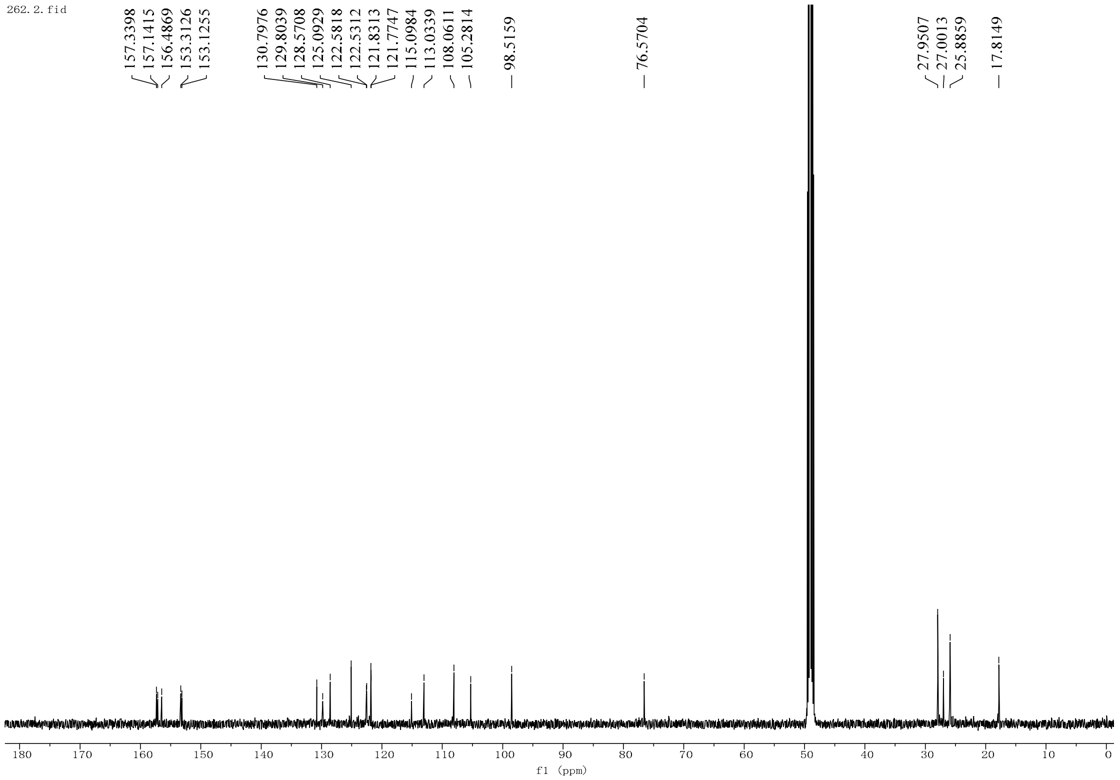

Figure S44. ^{13}C-NMR spectrum of compound **14** in CD_3OD
